# Responses of *Methanosarcina barkeri* to acetate stress

**DOI:** 10.1186/s13068-019-1630-5

**Published:** 2019-12-16

**Authors:** Pinjing He, Haowen Duan, Wenhao Han, Yang Liu, Liming Shao, Fan Lü

**Affiliations:** 1 State Key Laboratory of Pollution Control and Resource Reuse, Tongji University, Shanghai, 200092 China; 2 Shanghai Institute of Pollution Control and Ecological Security, Shanghai, 200092 China; 3 Institute of Waste Treatment and Reclamation, Tongji University, Shanghai, 200092 China

**Keywords:** *Methanosarcina barkeri* MS, Transcriptomic, Proteomic, Acetate stress, Anaerobic digestion

## Abstract

**Background:**

Anaerobic digestion of easily degradable biowaste can lead to the accumulation of volatile fatty acids, which will cause environmental stress to the sensitive methanogens consequently. The metabolic characteristics of methanogens under acetate stress can affect the overall performance of mixed consortia. Nevertheless, there exist huge gaps in understanding the responses of the dominant methanogens to the stress, e.g., Methanosarcinaceae. Such methanogens are resistant to environmental deterioration and able to utilize multiple carbon sources. In this study, transcriptomic and proteomic analyses were conducted to explore the responses of *Methanosarcina barkeri* strain MS at different acetate concentrations of 10, 25, and 50 mM.

**Results:**

The trend of OD600 and the regulation of the specific genes in 50 mM acetate, indicated that high concentration of acetate promoted the acclimation of *M. barkeri* to acetate stress. Acetate stress hindered the regulation of quorum sensing and thereby eliminated the advantages of cell aggregation, which was beneficial to resist stress. Under acetate stress, *M. barkeri* allocated more resources to enhance the uptake of iron to maintain the integrities of electron-transport chains and other essential biological processes. Comparing with the initial stages of different acetate concentrations, most of the genes participating in acetoclastic methanogenesis did not show significantly different expressions except *hdrB1C1*, an electron-bifurcating heterodisulfide reductase participating in energy conversion and improving thermodynamic efficiency. Meanwhile, *vnfDGHK* and *nifDHK* participating in nitrogen fixation pathway were upregulated.

**Conclusion:**

In this work, transcriptomic and proteomic analyses are combined to reveal the responses of *M. barkeri* to acetate stress in terms of central metabolic pathways, which provides basic clues for exploring the responses of other specific methanogens under high organics load. Moreover, the results can also be used to gain insights into the complex interactions and geochemical cycles among natural or engineered populations. Furthermore, these findings also provide the potential for designing effective and robust anaerobic digesters with high organic loads.

## Background

Anaerobic digestion (AD) has been highlighted as an important organic waste treatment technology for bioenergy production. Acetate is a main intermediate during AD processes and can be accumulated to high concentrations, due to the rapid fermentation of easily biodegradable waste [1]. The high acetate concentration is identified as a significant cause of inhibition in AD processes [2–4]. And it leads AD to a dilemma called “inhibited pseudo-steady state” along with ammonium and pH, where AD processes maintain stable performances under neutral conditions but with lower methane yields [5, 6]. Nevertheless, few studies provide direct insights into the responses of specific methanogens to acetate stress, which frequently occurs in engineered AD processes.

*Methanosarcina* can be dominant during AD processes [7–9] and are identified as the key functional microbes to alleviate acid inhibition [10]. They prefer a higher acetate concentration [11], maintain a stable acetate utilization rate [12, 13] and even survive at a pH value of approximately 4.5 [14]. Moreover, *Methanosarcina* can form complex multicellular structures to resist stress [15].

In addition to the dominant niche in AD processes, *Methanosarcina* has more advantages over other methanogens. It can yield methane through all four known methanogenesis pathways [16], and shift pathway according to the levels of stress [17–19]. Meanwhile, it has two disparate energy-conserving systems [20] and can alter electron transport chains [21, 22]. Additionally, *M. barkeri* has a much higher growth yield on H_2_ and CO_2_ than the methanogens without cytochromes [23]. Previous experiments indicated that Methanosarcinaceae in mixed consortia shifted methanogenesis pathways gradually. It was shifted from acetoclastic methanogenesis to hydrogenotrophic methanogenesis, when acetate concentrations increased from 50 to 150 and 250 mM [5]. All of these features determine the widespread distribution of *Methanosarcina* in AD processes and its high tolerance to acetate stress. Nevertheless, to the best of our knowledge, the responses of *Methanosarcina* to acetate stress are still unexplored.

Currently, omics have been widely implemented in the studies of AD processes to search for novel microorganisms [24], elucidate key metabolic activities [25] and other objectives [26]. Indeed, extensive studies on AD processes have made use of cultivation-independent biotechnologies to document the variations of microbial populations under acetate stress. But it is still difficult to gain the insights into the responses of specific methanogens, due to environmental complexities and microbial interactions in mixed consortia. In contrast, transcriptomic or proteomic analysis on pure cultivation can exclude interferences from other microbes, which therefore has been applied to explore the stress responses of specific microbes [27–29]. Nevertheless, the effective analytical method of combining pure cultivation with transcriptomic and proteomic analyses, has not yet to be used to explore the responses of specific methanogens to acetate stress.

Therefore, this analytical method was used in the present study to explore the responses of *M. barkeri* strain MS, a model methanogen, to different levels of organic load stresses formed by different total acetate concentrations. Regulations of the genes participating in stress response, signal transduction, element translocation, acetoclastic methanogenesis and nitrogen fixation, show the responses of *M. barkeri* to acetate stress, which provides bases to understand the responses of other methanogens. These findings are conductive to explore the complex interactions in mixed consortia during AD processes and develop strategies to alleviate inhibitory factors and optimize AD processes.

## Results

### The biochemical data and micrographs

**Fig. 1:**
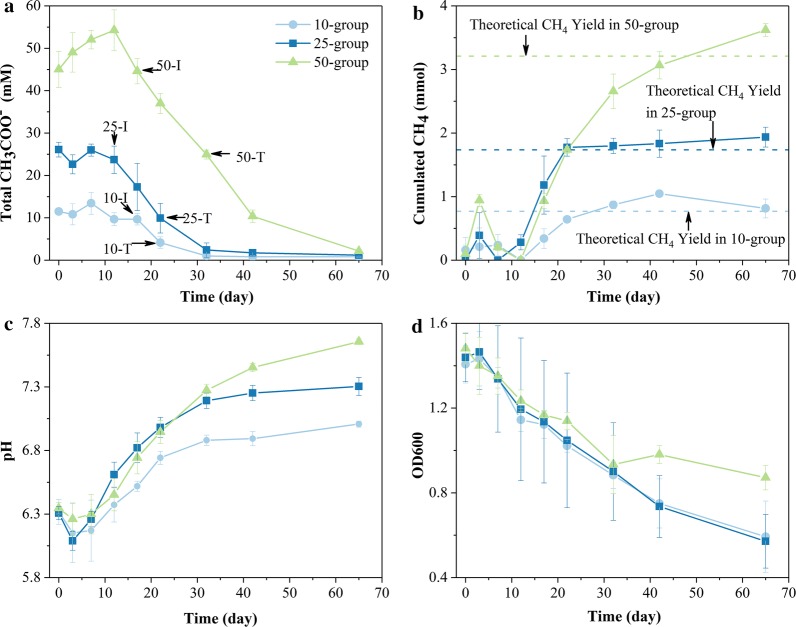
The biochemical indictors under different levels of acetate stress. **a** CH_3_COO^−^ concentration and sampling points. **b** Cumulated CH_4_ yield. **c** pH values in culture media. **d** OD600. Error bars indicate ± sd of biological triplicates; invisible error bars indicate the sd values are very small

The sampling points are marked in Fig. 1a. The 10-, 25-, and 50-group are labeled as “10”, “25”, and “50”, respectively. “I” and “T” represent initial and terminal sampling points, respectively. The methanogenesis started to proceed rapidly from the initial sampling points. The terminal sampling points can be used to verify under the condition that the total acetate concentration is approximately equal, whether the gene regulations of *M. barkeri* will be different after long-term inhibition. As shown in Fig. 1a, the total acetate concentrations of 25-T and 10-I, 50-T and 25-I were approximately equal, respectively.

The previous work suggested that free acetic acid functioned not only as the substrate for acetoclastic methanogenesis but also as an inhibitor [12], while present work was mainly to focus on the inhibitory effects of different acetate concentrations on *M. barkeri*. Both of the total acetate and free acetic acid concentrations showed significant differences among the initial sampling points (*P* < 0.05) (Additional file 1: Table S1), which indicated that the *M. barkeri* in three groups were undergoing different levels of acetate stress.

The trend of each biochemical data was fitted using the Boltzmann function (Additional file 2: Fig. S1 and Additional file 3: Table S2). The rates at each sampling point were calculated by the fitted curves (Table 1). During a lag phase of 10 days (Fig. 1a), the increase in acetate concentrations might be due to the gradual release of the acetate absorbed by cell aggregations. After the lag phase, acetate in three groups began to be degraded and was close to the utilization threshold, which is consistent with previous studies [30]. Methane was yielded simultaneously in the three groups. The accumulative yields of methane were roughly close to the theoretical yield of acetoclastic methanogenesis, with a maximum deviation of 12.9% (Fig. 1b). Along the degradation of acetate, the pH value in each group constantly increased from moderately acidic to neutral (Fig. 1c). The OD600 values decreased apparently and showed coherent descending trends in three groups during the first 30 days. But the descending trend in the 50-group was more gradual than that in the 10- and 25-group after the turning point (Fig. 1d and Table 1). Even though *M. barkeri* was no longer in exponential phase, the stress from stationary phase could not make OD600 values decrease so apparently, and the descending trend would not slow down or even cease. These characteristics indicated that acetate stress was absolutely the major stress and the *M. barkeri* in 50-T might have acclimated to the acetate stress. The latter was further proved by the higher minimal acetate consumption rate and minimal CH_4_ production rate in the 50-group than those in the 10- and 25-group (Additional file 4: Table S3).

**Table 1:** The biochemical data at the initial and terminal sampling points of three groups

Group	Sample point	OD600	OD600 descending rate (day^−1^)	Total acetate (mM)	Acetate consumption rate (mM/day)	CH_4_ production rate (mmol/day)	pH	pH rising rate (day^−1^)
10-group	10-I	1.122	0.018	9.66	0.73	0.049	6.47	0.046
	10-T	1.022	0.017	4.15	0.94	0.073	6.69	0.037
25-group	25-I	1.194	0.020	23.70	0.59	0.075	6.56	0.051
	25-T	1.048	0.018	9.92	1.57	0.034	6.93	0.036
50-group	50-I	1.168	0.015	44.59	0.74	0.095	6.69	0.040
	50-T	0.934	0.0079	24.93	1.78	0.092	7.22	0.030

As acetate concentration increased, cell aggregations disintegrated, with larger distances between cells, and irregular cell features instead of plump sphericity appeared (Additional file 5: Fig. S2a–c). It was difficult to find denser cell aggregations in 50-I, but cell aggregations of approximately 50 μm in length were observed in 10-I and 25-I (Additional file 5: Fig. S2m, n). It indicated that the high level of acetate stress interfered with the formation and stability of cell aggregations. The decrease in fluorescence signal of coenzyme F_420_ indicated that the activities of *M. barkeri* were weakened with the increase in acetate stress (Additional file 5: Fig. S2d–f). At the terminal sampling points, not only the size of cells themselves, but also the size of cell aggregations decreased, and many separate cells appeared. Cell features were generally irregular at the terminal sampling points (Additional file 5: Fig. S2g–i). The fluorescent signals, which were not observed in 10-T and 25-T, but were observed in 50-T. It indicated the active biological processes in *M. barkeri* (Additional file 5: Fig. S2j–l) and confirmed the OD600 trend in the 50-group (Fig. 1d).

### Transcriptomic analysis

**Fig. 2:**
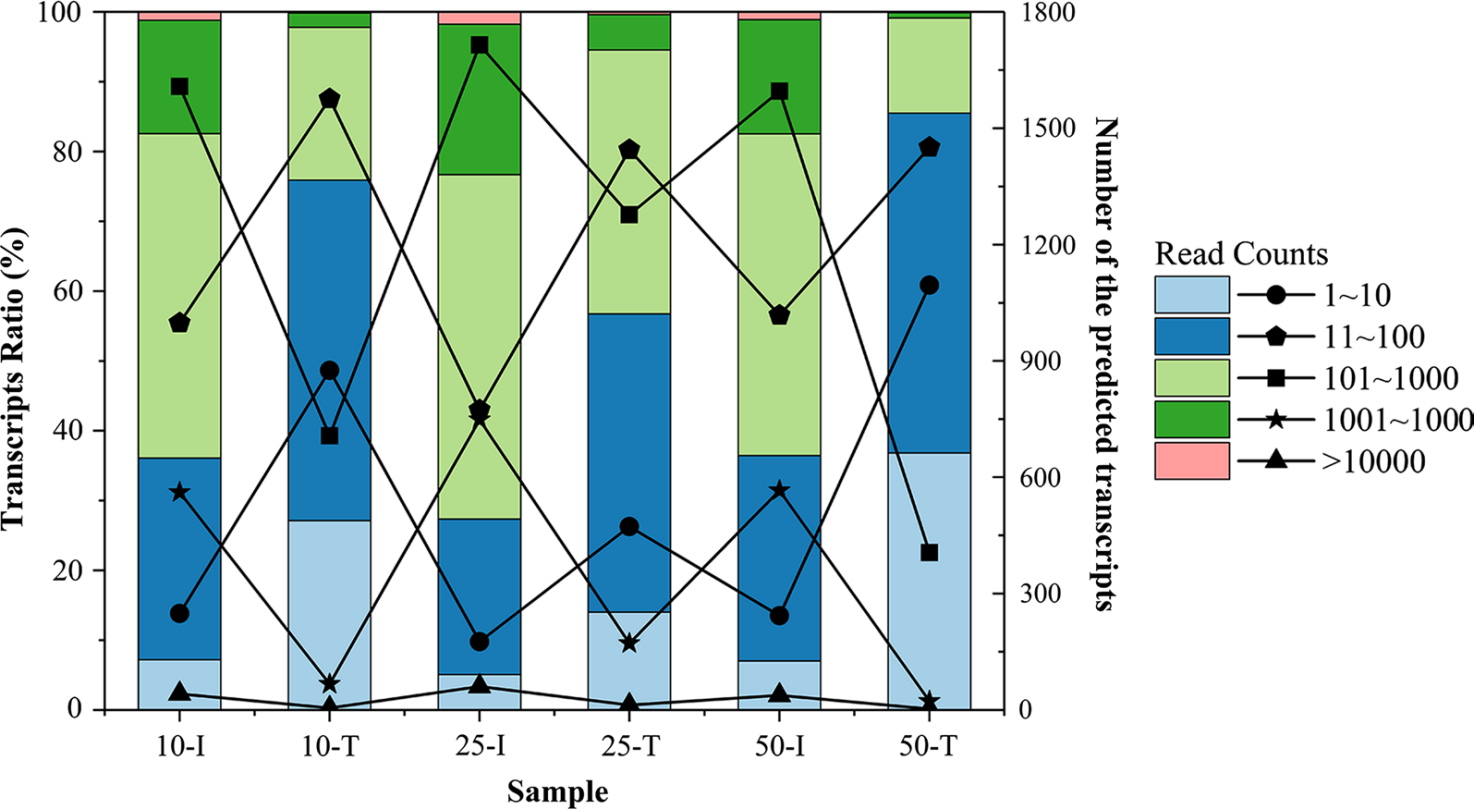
Ratio and number of predicted transcripts with different read counts in each sample. The bar chart represents the ratio of the transcripts with different read counts in the specified sample, and the line chart represents the number of these transcripts

The transcriptomic protocol yielded an average of 18,130,832 filtered non-rRNA reads with an average length of 145 bp per library (Additional file 6: Table S4a). Approximately 85.26–99.46% of the 3494 annotated open reading frames were recovered by transcriptomic analysis. As the reactions progressed, the proportions of high-abundance transcripts decreased with the increasing proportions of low-abundance transcripts in each group, indicating that the number of highly expressed genes decreased (Fig. 2).

The detailed information of all differently expressed genes (DEGs) is listed in Additional file 7: Dataset S1. There were more DEGs in “10-T_10-I”, “25-T_25-I”, and “50-T_50-I” than in “25-I_10-I”, “50-I_10-I”, and “50-I_25-I” (Additional file 8: Fig. S3), which illustrated that the regulation mechanisms during the consumption of acetate were more complex than these between the initial sampling points. The ratio of the identical DEGs among “10-T_10-I”, “25-T_25-I”, and “50-T_50-I” was up to 30%, and most of them were identified as hypothetical proteins, indicating that hypothetical proteins might be essential to resist acetate stress. The results of RT-qPCR indicated that the RNA-Seq data were reliable (Additional file 6: Table S4b).

**Fig. 3:**
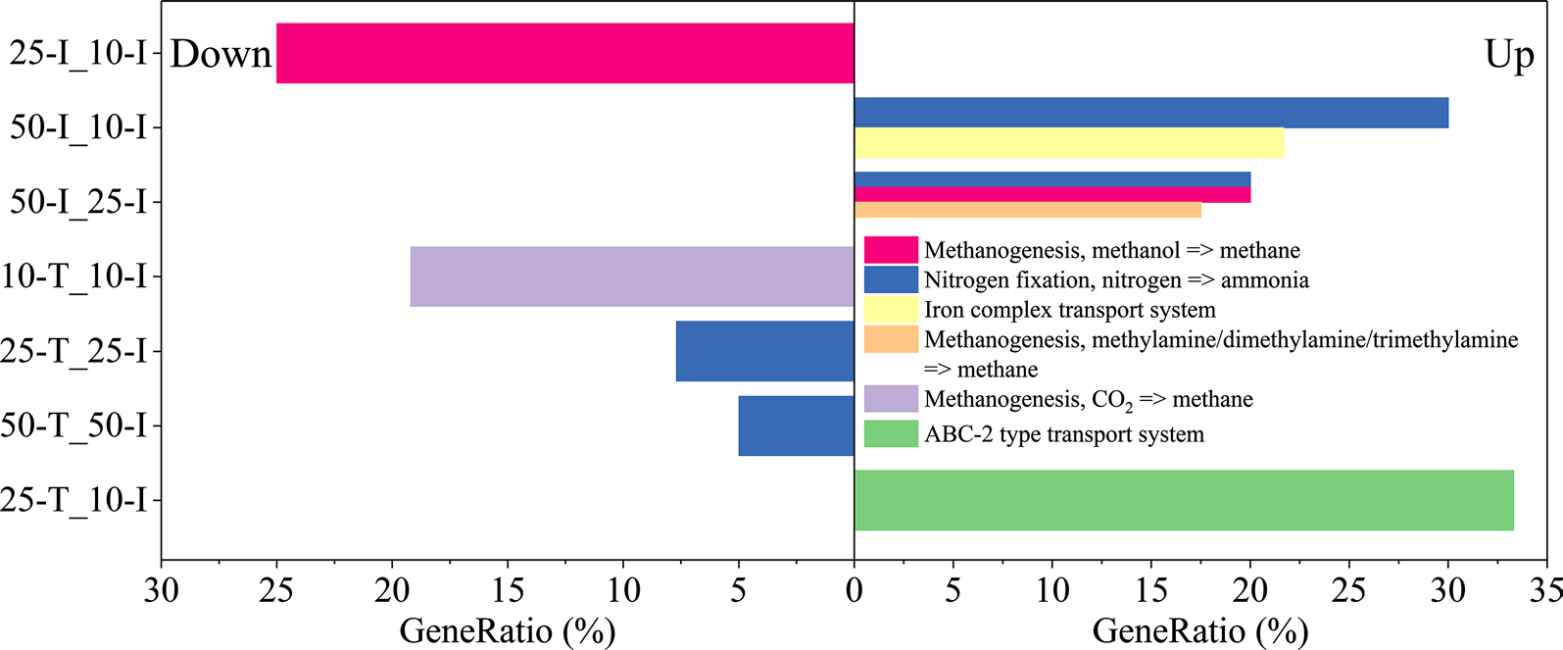
KEGG module enrichment analyses. The downregulated modules are shown on the left, and the upregulated modules are shown on the right. The filter criterion for KEGG module enrichment was *P* < 0.05 (Benjamini and Hochberg methods). Gene ratio is calculated by dividing k by n, where k is the number of DEGs assigned to the specific KEGG module and n is the number of DEGs annotated by KO identifiers

The KEGG module enrichment analysis showed that nitrogen fixation pathway was upregulated significantly in “50-I_10-I” and “50-I_25-I”. With the consumption of acetate, nitrogen fixation pathway was downregulated in “25-T_25-I” and “50-T_50-I” (Fig. 3). Nevertheless, there was no significant up- or downregulation in acetoclastic methanogenesis pathway, which was the main methane metabolism using acetate as substrate.

**Fig. 4:**
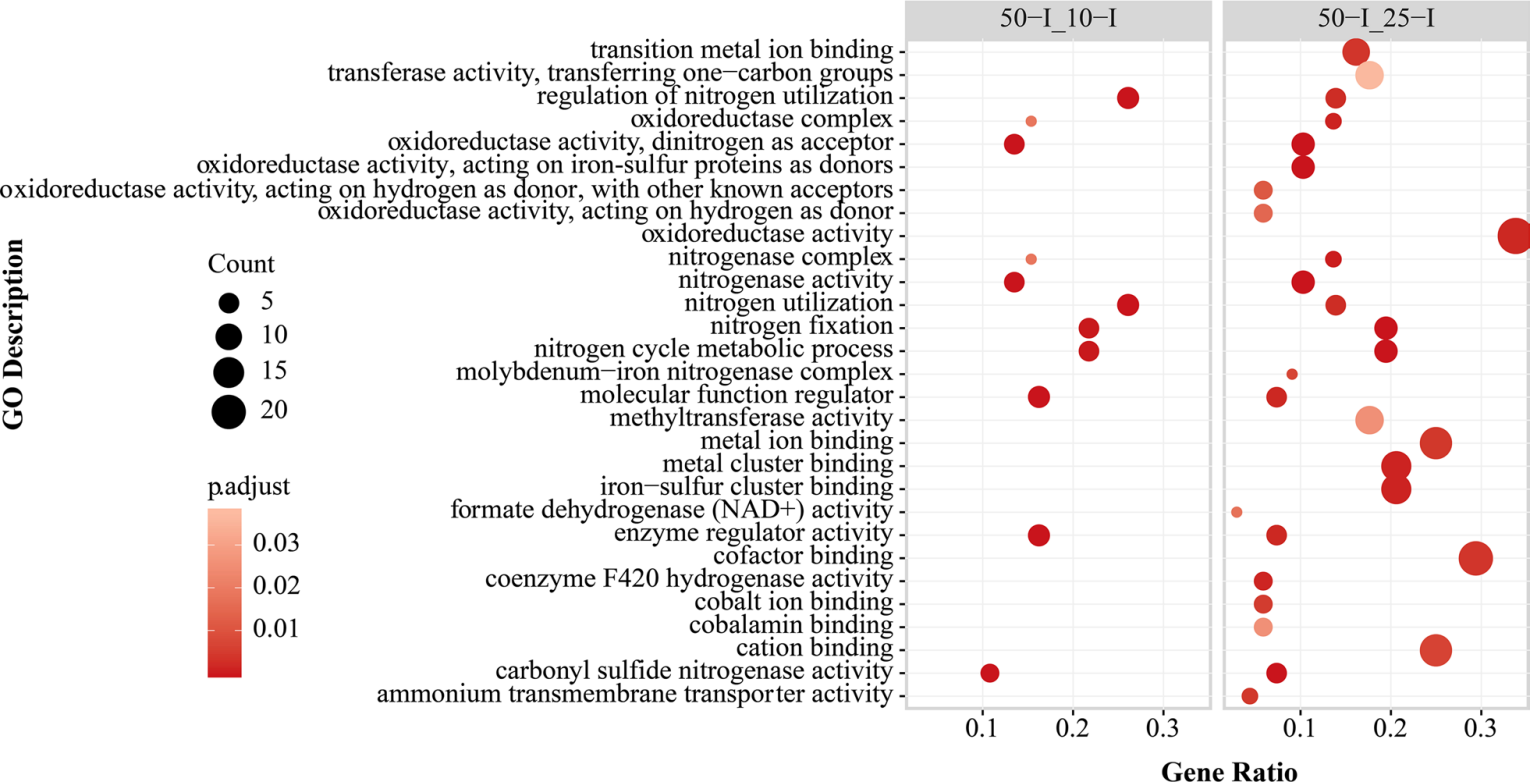
GO enrichment analyses of upregulated DEGs in “50-I_10-I” and “50-I_25-I”. The significance of GO enrichment analyses was calculated by Benjamini and Hochberg method

**Fig. 5:**
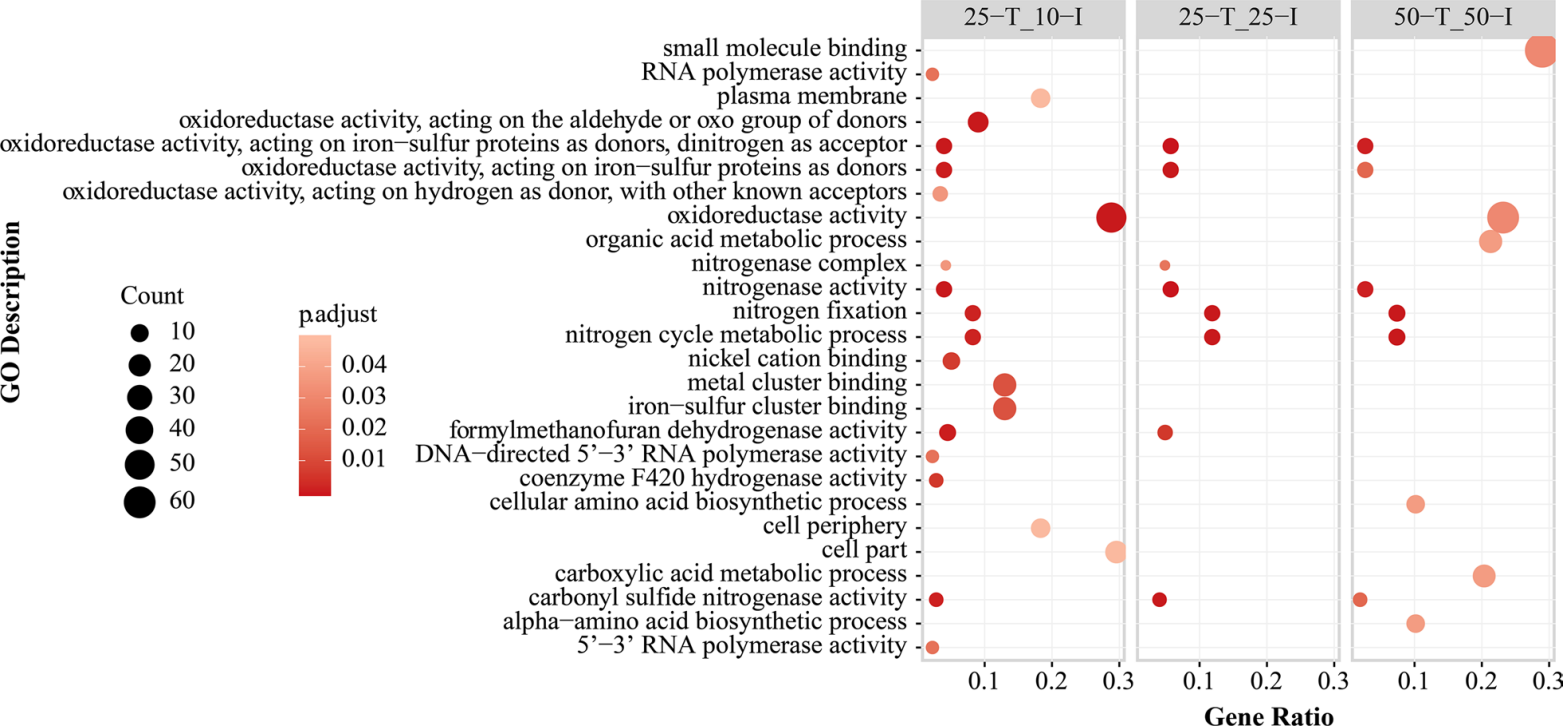
GO enrichment analyses of downregulated DEGs in “25-T_10-I”, “25-T_25-I”, and “50-T_50-I”. The significance of GO enrichment analyses was calculated by Benjamini and Hochberg method

For the comparisons between the initial sampling points, the GO enrichment analysis also showed that the terms related to nitrogen fixation and nitrogen utilization were upregulated in “50-I_10-I” and “50-I_25-I” (Fig. 4), which were consistent with the results from above KEGG module enrichment analysis. The activities related to quorum sensing (QS) in “50-I_25-I”, such as response to stimulus and cell communication, were downregulated (Additional file 9: Fig. S4). With the consumption of acetate, the nitrogenase activities in “25-T_25-I” and “50-T_50-I” were downregulated (Fig. 5).

WGCNA identified 13 modules of genes and the minimum module size was 32 transcripts. These modules were distinguished by colors (Additional file 10: Dataset S2). 100 and 115 genes (labeled as “N_Ac” and “N_pH” groups, respectively) were negatively correlated with acetate consumption rates and pH values, respectively, of which 56 genes were identical (Additional file 10: Dataset S2). These genes might play essential roles at the initial sampling points where inhibitory factors were the strongest. Gene MSBRM_0367 including both the “N_Ac” and “N_pH” groups encodes transcriptional regulators, associated with the genes participating in nitrogen metabolism, QS, methane metabolism and other processes (Additional file 11: Fig. S5a). In addition to gene MSBRM_0367, gene MSBRM_0203 and MSBRM_2051 were also included in the “N_pH” group, and these two genes had relationships with the genes participating in energy synthesis, transcriptional regulation, sensory transduction, iron uptake and other processes, indicating that these processes were essential for resisting stress at the initial sampling points (Additional file 11: Fig. S5c, d). The detailed results of WGCNA are described in Additional file 12: Text S1.

### Proteomic analysis

**Fig. 6:**
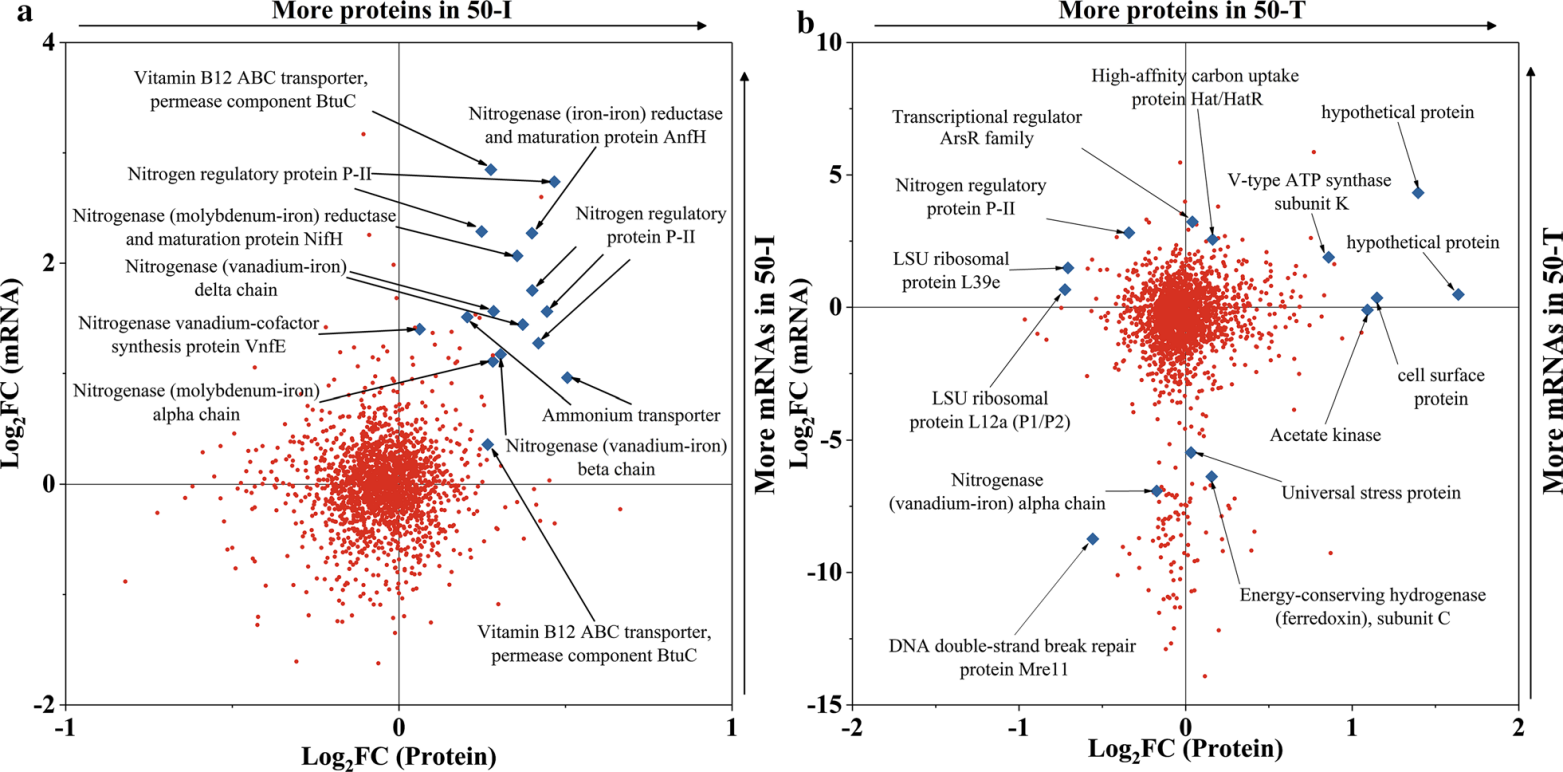
The fold changes in mRNAs and their corresponding proteins. **a** “50-I_10-I; **b** “50-T_50-I”. Red dots or blue diamond points represent genes detected in both transcriptomic and proteomic

Proteomic analysis recovered 1813 of 3494 predicted proteins. In “50-I_10-I”, there were 29 and 54 proteins showing upregulation and downregulation, respectively (Additional file 13: Dataset S3). In “50-T_50-I”, 144 and 68 proteins showed upregulation and downregulation, respectively (Additional file 13: Dataset S3). The fold changes in mRNAs and their corresponding proteins in “50-I_10-I” or “50-T_50-I” did not show coherence (Fig. 6), indicating that transcription and translation were uncoupled for most proteins. In “50-T_50-I”, the proteins with the largest fold changes were hypothetical proteins, and approximately 33% of the differently expressed proteins (DEPs) were hypothetical proteins. The high proportion of hypothetical proteins revealed huge gaps in the knowledge of the responses to acetate stress. In “50-I_10-I”, the mRNAs and their corresponding proteins participating in nitrogen fixation, were all upregulated, which highlighted the importance of bioavailable nitrogen sources for resisting acetate stress (Fig. 6a).

In “50-I_10-I” and “50-T_50-I”, most of the DEPs that were identified as hypothetical proteins were cytoplasmic proteins, indicating they rarely participated in transmembrane processes like substance transport. These proteins might primarily participate in cytoplasmic activities to maintain the internal stabilities of cells. For the hypothetical proteins that were identified as signal peptides, most of them could not be located (Additional file 13: Dataset S3), showing complex signaling networks under acetate stress. Even if the above results showed the relationships of these hypothetical proteins to acetate stress, it was problematic to speculate their detailed functions.

## Discussion

### Stress proteins under different levels of acetate stress

The genes encoding heat shock proteins (Hsp 60) and DNA double-strand break (Dsb) repair proteins were upregulated in “50-I_25-I” and “50-I_10-I”, respectively. These proteins participated in mitochondrial protein import or macromolecular assembly and restrained cell death or comprehensive genetic variation in cells. The ATP-dependent DNA ligase gene (*lig1*) was also upregulated in “50-I_10-I” and “50-I_25-I”, which has an active role in DNA replication, recombination and repair [31]. This indicated that the DNA structure was unstable at the initial sampling point under high level of acetate stress, although *M. barkeri* was capable of surviving in 50 mM acetate [12, 32].

Along with the consumption of acetate, most of the DEGs encoding stress proteins and Lig1 were upregulated in “10-T_10-I”, indicating that *M. barkeri* still did not acclimatize to the acetate stress. Nevertheless, in “50-T_50-I”, most of the genes encoding stress proteins were downregulated according to the transcriptomic analysis, and proteomic analysis showed that all detected Hsp 60 and Dsb proteins were downregulated. In “50-T_25-I”, the *dsb* was downregulated. Furthermore, most of the DEGs encoding Hsp, Dsb and universal stress proteins were upregulated in “25-T_10-I”. Considering the OD600 trend in each group (Fig. 1d) and the regulation trends of DEGs encoding stress proteins, it was revealed that a period of exposure to a high level of acetate stress might promote *M. barkeri* to acclimatize to acetate stress. This presumption was consistent with the phenomenon in our previous study where the abundance of Methanosarcinaceae started to be detected or increase in the later stages of the reactions [5].

### Signal transduction in cell aggregations

The genes encoding cell division proteins (FtsEX complex) which were essential for assembling or stabilizing the septal ring [33], were downregulated in “10-T_10-I” and “25-T_25-I” (6.41-fold and 13.27-fold, respectively). Nevertheless, these genes showed a slightly decreased fold change in “50-T_50-I” (2.06-fold). These regulation trends roughly coincided with the OD600 trends (Fig. 1d). *ftsH* participates not only in cell division and cell control, but also in membrane functions and gene expression, which was upregulated in “50-T_25-I” and downregulated in “25-T_10-I”. Logically speaking, a low level of stress had fewer negative impacts on cells, but the present work indicated that a high level of acetate stress could stimulate the greater “potential” for *M. barkeri* to resist stress.

Cell proliferation could facilitate the formation of cell aggregations. The micrographs and the previous study found that *M. barkeri* resisted stress by forming multicellular aggregations (Additional file 5: Fig. S2) [34]. In the cell aggregations, the gradients of acetate concentrations were formed to alleviate the acetate stress on the inner cells and prevent more H_2_ from diffusing [35]. H_2_ cycling was essential for acetoclastic methanogenesis [36].

Cell aggregations exchanged information of cell densities by quorum sensing (QS) system using peptide signals, such as oligopeptides and dipeptides. In “25-I_10-I”, “50-I_10-I”, and “50-I_25-I”, the genes encoding periplasmic oligopeptide-binding protein (OppA) or oligopeptide/dipeptide transport system permease proteins (OppC and DppB) were upregulated. Nevertheless, the genes encoding ATP-binding proteins (OppD and DppF) were downregulated in “25-I_10-I” and “50-I_10-I”. ATP hydrolysis was essential for the normal functions of ABC transport system [37], therefore, the downregulation of *oppD* and *dppF* indicated that acetate stress could block the information exchange on a community-wide scale. The blocked information exchange offset the advantages of cell aggregations, and *M. barkeri* might not make proper responses to acetate stress. Because the activation of the Type II secretion system proteins was under the control of QS system [38], the downregulation of the DEGs encoding Type II secretion system proteins also indicated the blocked information exchange. In other comparisons, *opp*/*dppBCDF* were all downregulated, but *opp/dppA* did not show up- or downregulation. It indicated that *opp/dppA* was more resistant to acetate stress, which was helpful to maintain the normal functions of the QS system in cell aggregations.

Approximately half of the DEGs encoding LSU and SSU ribosomal proteins were upregulated in “50-T_50-I” and “50-T_25-I” (41.4% and 55.6%, respectively), but 88.9% of the DEGs encoding the same proteins were downregulated in “25-T_10-I”. The expression of ribosomal proteins usually represented protein synthesis, which was an energy-consuming process accounting for 75% of energy expenditure in cells [39]. It was revealed that *M. barkeri* gradually recovered energy synthesis to support the synthesis of some proteins in the 50-group, which helped *M. barkeri* acclimatize to acetate stress.

### Membrane and ABC transporters for translocating elements

Transmembrane transfer activities were related to membrane and ABC transporters. Except for the comparisons between the initial sampling points, the fluctuant regulation trends of the DEGs encoding cell surface proteins in other comparisons indicated that the responses of cell membranes were complicated. And in “10-T_10-I”, “25-T_25-I”, “50-T_50-I”, and “50-T_25-I”, the genes encoding mannose-6-phosphate isomerase were upregulated, which was related to polysaccharide biosynthesis and the modification of cell wall carbohydrates. It indicated that *M. barkeri* modified its cell surface structures according to the levels of acetate stress, which was also observed in the previous study [40].

The phosphate transport system (Pst) was a more efficient system for Pi translocation in *M. barkeri*. In “10-T_10-I” and “25-T_25-I”, *pstB* was downregulated which provided energy to free Pi [41]. It indicated that acetate stress could result in an insufficient energy supply for translocating elements and thereby cause a series of negative effects on physiological functions. But *pstB* did not show significant regulation in “50-T_50-I” and “50-T_25-I”, therefore the stable expression of *pstB* might promote the acclimation of *M. barkeri* to the acetate stress in 50-group.

Iron is essential for maintaining stable methanogenesis [42, 43], where iron was a main component in redox enzymes, such as ferredoxin and Hdr. The previous studies demonstrated that iron deficiency hindered nucleotide synthesis and ATP production [44, 45]. For more iron uptake, the genes encoding ferrous iron transport protein B, which were essential for iron supply in anaerobic conditions, were upregulated in “50-I_10-I” and “50-I_25-I”. And the proteomic analysis also showed the upregulation of iron complex transport system permease proteins in “50-I_10-I” (Additional file 13: Dataset S3). Furthermore, the iron complex transport activity was enriched in “50-I_10-I” (Fig. 3). It was suggested that the reason why exogenous iron could maintain stable methanogenesis was not only it could enhance the interspecies electron transfer between species and reduce oxidative-reductive potential [46, 47], but also it could maintain the integrities of some key enzymatic activities. Compared with the gene regulation related to other elements’ uptake, the upregulation of iron uptake indicated the inhibited *M. barkeri* would allocate more resources to enhance the uptake of more essential elements, especially under high level of acetate stress.

The inappropriate transmembrane ion gradients formed under acetate stress could cause the imbalance of osmotic pressures. The glycine betaine transport system (Pro) could provide osmo-protection [48]. In “25-I_10-I” and “50-I_10-I”, *proV* encoding ATP-binding protein was downregulated. The insufficient energy supply would hinder the normal functions of Pro under acetate stress, which thereby induced cell death. The upregulation of *proV* in “50-T_25-I” also might promote the acclimatization of *M. barkeri* to the acetate stress in 50-group. As expected, *proV* was downregulated in “25-T_10-I”, where the *M. barkeri* did not acclimatize to the acetate stress.

### Acetoclastic methanogenesis for energy synthesis

**Fig. 7:**
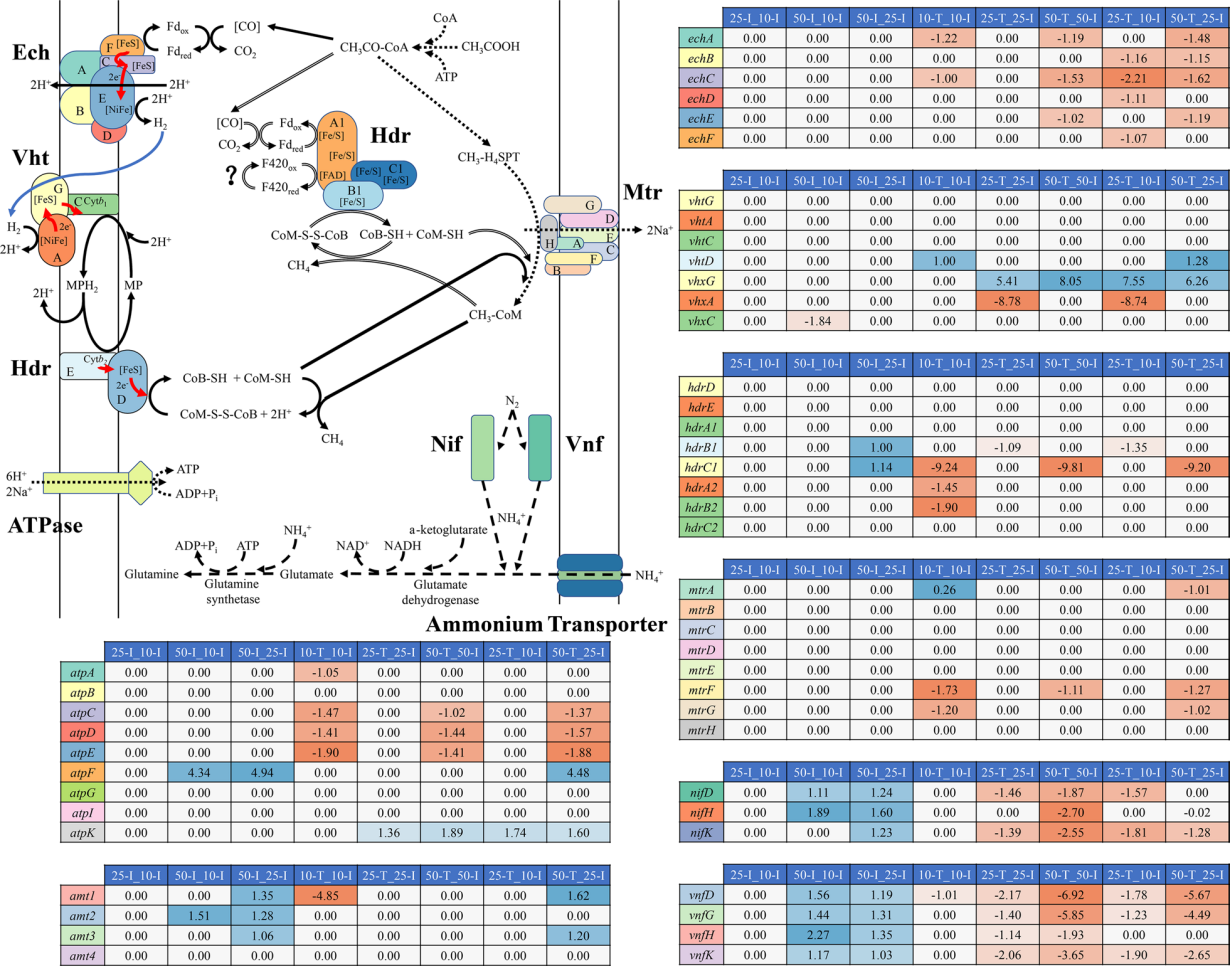
The transcriptional changes in genes involved in proposed acetoclastic methanogenesis, nitrogen fixation and ATP synthesis by different comparisons. The values in the heatmaps show the average Log_2_ fold changes in transcripts translating the same subunit of each enzyme in different comparisons. Red represents downregulation, blue represents upregulation, and white represents no significant regulation. The solid lines indicate the conventional acetoclastic methanogenesis. The double bond lines indicate the postulated acetoclastic methanogenesis. The dotted lines indicate the overlap of conventional acetoclastic methanogenesis and postulated acetoclastic methanogenesis. The dashed lines indicate the nitrogen fixation pathway. Red solid lines indicate electron flow in each enzyme and blue solid lines indicate H_2_ flow in acetoclastic methanogenesis. The question mark represents the enzyme re-oxidizing F_420_H_2_

As the terminal of electron transfer, methane was yielded through acetoclastic methanogenesis with energy synthesis. In “50-I_25-I”, the genes encoding heterodisulfide reductase subunits (HdrB1C1) were upregulated, but the *hdrDE* did not show significant regulations. HdrA1B1C1 had been speculated to be an electron-bifurcating enzyme in energy conversion as shown in Fig. 7. One possible explanation for why multitype Hdr expression was that HdrABC could help cells to acclimatize to the fluctuations in substrate concentrations and conserve energy more efficiently [23, 49]. The available free energy from acetoclastic methanogenesis under standard condition (Δ*G*^o^*ʹ* = − 36 kJ/CH_4_) provided only a small amount of energy for growth, because of the endergonic reaction where acetate was activated to acetyl-CoA. The upregulation of *hdrB1C1* could be a complementary mechanism for *M. barkeri* to improve the thermodynamic efficiency under the high level of acetate stress.

*Methanosarcina barkeri* was one of the few methanogens that could participate in direct interspecies electron transfer [50], and it could shift methanogenesis pathways according to stress levels. Furthermore, *Methanosarcina* could produce H_2_ and act as a syntrophic acetate oxidizer during growth on acetate [32]. On the other hand, HdrA1B1C1 and homologous HdrA2B2C2, which had similar functions [49, 51], were suggested to be essential for direct interspecies electron transfer [52]. These characteristics indicated there might be a preliminary hypothesis that in the cell aggregations formed under high level of acetate stress, some *M. barkeri* might act as syntrophic acetate oxidizers, and another *M. barkeri* might perform hydrogenotrophic methanogenesis to yield more energy for cellular activities. Therefore, electron transfer would exist in one species with distinct pathways. Moreover, the DEGs encoding coenzyme F_420_ hydrogenase subunit alpha, beta, and gamma (FrhABG), which participated in hydrogenotrophic methanogenesis, were upregulated in “50-I_25-I”. The upregulation of *frhABG* was part of the evidence for the electron transfer between *M. barkeri* under high level of acetate stress. And the hypothetical proteins occupying most of the proteome might also participate in this electron transfer. Nevertheless, this hypothesis needs to be further verified.

The other key genes participating in acetoclastic methanogenesis did not show significant regulation at the initial sampling points (Fig. 7). It indicated that *M. barkeri* metabolized acetate stably under different levels of acetate stress within its tolerance range. The stable acetate metabolism could help *M. barkeri* to outcompete other methanogens for acetate and thereby dominating mixed consortia. The gene encoding V-type ATP synthase subunit F (AtpF), which was likely a regulatory subunit for controlling pH homeostasis, was upregulated in “50-I_10-I” and “50-I_25-I” (Fig. 7). Therefore, maintaining pH homeostasis was a mechanism of *M. barkeri* to hold the intracellular environment suitable under acetate stress.

In “10-T_10-I”, with the reactions proceeding, *hdrA2B2C1* were downregulated (Fig. 7). And the genes encoding energy-conserving hydrogenase subunits A and C (EchA and EchC, respectively), which are essential for forming transmembrane proton gradients and keeping H_2_ cycling during acetoclastic methanogenesis, were also downregulated (Fig. 7). Furthermore, the gene encoding tetrahydromethanopterin S-methyltransferase subunit A (MtrA) was downregulated, which could form a transmembrane sodium gradient for ATP production [16, 21]. Meanwhile, *atpAECD* were downregulated. These results indicated that an insufficient acetate concentration had negative impacts on the energy synthesis of *M. barkeri*, whose affinity for acetate was low. It might be one of the reasons why *M. barkeri* were eliminated in low acetate concentrations. Although *mtrFG* were also downregulated, they appeared to be the nonessential components in *mtr* operon in terms of transferring methyl [53]. The same regulation trends of *mtrAFG* indicated that there might have potential interactions among them.

In “25-T_25-I”, methanophenazine-reducing hydrogenase (VhxAG) with unknown functions, showed opposite regulation trends (up- and downregulation, respectively). Furthermore, *vhx* in “10-T_10-I”, “25-T_25-I”, and “50-T_50-I” showed significant regulation. It was suggested that the deletion of *vhxD* in *vhx* operon caused Vhx to have no analogous functions of another methanophenazine-reducing hydrogenase (Vht) [20, 54], which was essential for Hdr to conserve energy in acetoclastic methanogenesis [36]. Nevertheless, *vhx* operon could encode the main functional subunits homologous to Vht, and VhtD might process Vhx under the controls of the DEGs encoding mobile element proteins or transposons. Combining the gene regulations with the operon structure, it indicated that Vhx might participate in acetoclastic methanogenesis as a hydrogenase. In “50-T_50-I”, the downregulation of *ech* and *atp* might be caused by the less acetate concentration at “50-T” point where *M. barkeri* had acclimatized to the acetate stress as discussed above.

### Methanogenic N_2_ fixation for bioavailable nitrogen resources

Methanogenic N_2_ fixation is proposed as an essential way to provide bioavailable nitrogen resources [55], and the methanogens being capable of fixing N_2_ such as *M. barkeri, Methanobacterium bryantii* and so on, distribute in diverse habitats [56–58]. The DEGs encoding nitrogenase (molybdenum–iron) subunits (NifHDK), and alternative nitrogenase (vanadium–iron) subunits (VnfHDK), which has lower catalytic efficiency in *Methanosarcina* species [59], were upregulated in “50-I_10-I” and “50-I_25-I” (Fig. 7). It indicated that ammonia was indispensable for *M. barkeri* to resist acetate stress, which could also be confirmed by the upregulation of the DEGs encoding ammonium transporters and nitrogen regulatory protein p II. In “50-I_10-I”, the regulation trends of the nitrogen regulatory proteins, nitrogenases and ammonium transporters, showed positive correlation between the transcriptomic and proteomic analyses (Fig. 6a). Ammonia can be assimilated into cellular nitrogen by glutamine synthetase (Gs) and glutamate dehydrogenase (Gdh) (Fig. 7). Gs was identified as a DEP in “50-I_10-I”, and *gs* was upregulated in “50-I_25-I”.

Ammonia is a biologically useful reduced form incorporated into amino acids and other vital compounds. Therefore, the enhanced nitrogen fixation pathway could help *M. barkeri* maintain the normal functions of key biological processes in the initial stages of acetate stress. And it could also help the more sensitive *M. barkeri* to outcompete other methanogens under acetate stress, as observed in the previous studies [5, 34]. Although *M. barkeri* could grow under nitrogen fixation conditions with acetate, the growth rate was considerably slower than with ammonium as nitrogen source [60]. Therefore, ammonium was used as the nitrogen source in the present work and its concentration was about 9.35 mM as the recommended cultivation condition, which could support the normal growth of *M. barkeri*. The enhanced nitrogen fixation pathway indicated that more bioavailable nitrogen sources were essential to resist acetate stress. However, as another major problem of anaerobic digestion, ammonia stress is caused by the high concentration of ammonia. Therefore, it is necessary to find a “best of both worlds” ammonia concentration, which still needs further explorations.

In addition to maintaining the normal functions of key biological processes, the enriched nitrogen fixation pathway might also keep the proton balance in cells. Under the same pH values, acetate stress produces relatively more free acetic acid, which can diffuse into cells. The intracellular is a near-neutral environment, so free acetic acid will be dissociated and then increase the intracellular proton concentration. The excessive intracellular protons could uncouple proton force on cytomembrane and hinder energy synthesis [61, 62]. Nevertheless, the ammonia synthesized by the enriched nitrogen fixation pathway could bind the excessive protons in cytoplasm, and thereby alleviated the imbalance of protons, which was toxic to *M. barkeri*.

Except for “25-I_10-I”, “50-I_10-I”, and “50-I_25-I”, the DEGs encoding nitrogenase subunits were all downregulated in other comparisons. For “10-T_10-I”, “25-T_25-I”, and “25-T_10-I”, the downregulation might be caused by the insufficient energy synthesis as discussed above, because N_2_ fixation required abundant reducing power and ATP [55, 63]. For “50-T_50-I” and “50-T_25-I”, *M. barkeri* had acclimatized to the acetate stress at the “50-T” point, indicating more nitrogen sources were not needed.

### The differences between proteomic and transcriptomic analyses

The regulations of some mRNAs and their corresponding proteins showed poor correlation, which was well known and observed in previous studies [64, 65]. Proteins could remain stable even beyond the entire cell cycle relative to mRNAs, whose half-lives averaged only a few min in *E. coli* [66]. The different temporal scales of transcriptome and proteome could be used to explain the poor correlation between some mRNAs and proteins. In addition to the differences in temporal scales, posttranscriptional or translational regulations were another explanation. The low proportion of coding regions in the genome of *M. barkeri* (79.2%) indicated that a large number of antisense RNAs and sRNAs existed, which participated in posttranscriptional regulation. The modulation effects of sRNAs played a prominent role in archaea by influencing the evolution and stability of mRNAs and translation efficiency [65, 67]. Translation initiation also affected mRNA–protein correlation [67], therefore the DEGs encoding translation initiation factors in “50-T_50-I” could explain the poor mRNA–protein correlation in part.

## Conclusions

The present work describes the gene regulations of *M. barkeri* in terms of the main metabolism pathways under acetate stress using transcriptomic and proteomic analyses. The findings provide bases to understand responses of other methanogens, and are conductive to explore the complex interactions in mixed consortia. As the sole conductor of methanogenesis, methanogens have multifaceted impacts on mixed consortia.

The 50 mM acetate had the most severe inhibition on *M. barkeri* between the initial sampling points, while the high level of acetate stress (50 mM) promoted the acclimation of *M. barkeri* to stress. The information exchange by QS system was hindered by insufficient ATP hydrolysis, which offset the advantages of cell aggregations. Enriched iron uptake suggested that *M. barkeri* could allocate more resources to enhance the uptake of key elements relative to other elements. Therefore, the addition of external iron might be a useful approach to alleviate acetate stress in engineered anaerobic digestors.

As the sole pathway to metabolize acetate, the acetoclastic methanogenesis pathway did not show significant regulation of conventional enzymes between the initial sampling points. The high tolerance of acetoclastic methanogenesis to acetate stress made Methanosarcinaceae the dominant methanogens in mixed consortia, where the other microbes in the outer layer of granule sludge could further provide protection. The expressions of *hdrABC* were complementary mechanisms to keep H_2_ cycling and improve thermodynamic efficiency under acetate stress. It was widely considered that Vhx could not function as homologous Vht due to the lack of maturation protease, while the significant regulation of *vhx* revealed that Vhx might be a complement to Vht, which needs to be further explored.

The enriched in methanogenic N_2_ fixation pathway revealed that sufficient bioavailable nitrogen sources were essential for sensitive *M. barkeri* to resist acetate stress. However, the mechanisms where N_2_ fixation alleviated acetate stress needed to be further explored.

The genes encoding the different subunits of one complex did not show the same regulation trends. On the other hand, the genes encoding the same subunit had distinct regulation trends. Furthermore, a considerable proportion of the DEGs were annotated as hypothetic proteins, and many novel transcripts were assembled by the software. This indicated that comprehensive gene regulations existed in *M. barkeri* under acetate stress, and some genes were activated, which were not expressed under suitable conditions. Future studies can focus on the functions of the DEGs encoding hypothetic proteins, which may prevent the disorder of physiological activities and adjust pathways dynamically. Meanwhile, the complex interactions in mixed consortia under acetate stress are unclear and need to be further explored.

## Methods

All materials and methods are described in detail in Additional file 12: Text S1.

### Pure cultivation and stress conditions

*Methanosarcina barkeri* MS (DSM 800) was incubated in 250-mL sealed serum bottles with a 160-mL medium volume at 35 °C. These serum bottles were divided into three groups, named as 10-group, 25-group, and 50-group, in which the total acetate concentrations were set as 10, 25, and 50 mM, respectively (Additional file 14: Fig. S6). Although the highest acetate concentration used in the previous study was 250 mM [12], it far exceeded the actual acetate concentration in engineered AD processes (Additional file 15: Table S5), and also far exceeded the acetate concentration in some studies on pure methanogens [32, 68, 69]. Therefore, the highest acetate concentration in the present work was set as 50 mM, which was used in the previous study [32]. The present work mainly focused on the responses of *M. barkeri* to different total acetate concentrations, therefore the initial pH values of three groups were controlled at about 6. Acetoclastic methanogenesis could not proceed well below this pH value.

### Transcriptomic analysis

The raw reads were filtered and trimmed using fastp [70]. Trimmed reads were aligned to the reference genome from Ensembl Genomes database using HISAT2 [71]. Stringtie [72] was applied to quantify the abundances of mRNAs. DEGs were estimated by edgeR [73]. The selection criteria for DEGs was a fold change > 2 and false discovery rate < 0.05. The DEGs were verified by RT-qPCR analyses. The GO and KEGG module enrichment analyses of DEGs were conducted using clusterProfiler [74]. The weighted correlation network analysis was conducted using WGCNA [75]. The raw data have been deposited to NCBI in SRA (PRJNA528099).

The comparisons are labeled in the format of “sampling point 1_sampling point 2”, where sampling point 1 represents the treatment sample and sampling point 2 represents the control sample. The comparisons included “25-I_10-I”, “50-I_10-I”, “50-I_25-I”, “10-T_10-I”, “25-T_25-I”, “50-T_50-I”, “25-T_10-I”, and “50-T_25-I”.

### Proteomic analysis

The cell pellets collected from 10-I, 50-I, and 50-T were used for proteomic analysis. The digested peptides were labeled using iTRAQ reagents (AB Sciex). The labeled peptides were analyzed on an EASY-nLC 1200 system coupled to a Q-Exactive mass spectrometer (Thermo-Fisher). All raw data were collected using Thermo Xcalibur and analyzed using Sequest HT (Thermo-Fisher). Proteomic data have been deposited to the ProteomeXchange Consortium via the iProx partner repository with the dataset identifier PXD013207. The prediction of transmembrane helices were conducted by TMHMM [76]. PSORTb [77] was used to predict the proteins’ subcellular localization, and SignalP [78] was used to predict the presence of signal peptides.

## Supplementary information

**Additional file 1: Table S1.** The concentration of total acetate and free acetic acid in each group.

**Additional file 2: Fig. S1.** Fitted curves of biochemical data. (a) Total CH_3_COO^−^ concentration; (b) Cumulated CH_4_ yield; (c) pH values in culture media; (d) OD600 values. The curves were fitted using the Boltzmann function on the average values of the data at each sampling point.

**Additional file 3: Table S2.** The parameter values of each fitted curve.

**Additional file 4: Table S3.** Maximum and minimum rates of 10-, 25- and 50-group.

**Additional file 5: Fig. S2.** The brightfield and epifluorescence micrographs of *M. barkeri* MS from different sampling points. (a), (d), and (m) 10-I; (b), (e), and (n) 25-I; (c) and (f) 50-I; (g) and (j) 10-T; (h) and (k) 25-T; (i) and (l) 50-T.

**Additional file 6: Table S4.** The transcriptomic sequencing data analysis. (a) The counts of filtered non-rRNA reads mapped to the reference genome. (b) Fold changes of differentially expressed genes from RNA-seq and RT-qPCR in different comparisons.

**Additional file 7: Dataset S1.** The detailed information of all DEGs in each comparison.

**Additional file 8: Fig. S3.** The regulation of genes in each comparison. (a) “25-I_10-I”; (b) “50-I_10-I”; (c) “50-I_25-I”; (d) “10-T_10-I”; (e) “25-T_25-I”; (f) “50-T_50-I”; (g) “25-T_10-I”; (h) “50-T_25-I”. The number of DEGs doesn’t include the novel transcripts assembled by Stringtie.

**Additional file 9: Fig. S4.** GO enrichment analysis of downregulated DEGs in “25-I_10-I” and “50-I_25-I”.

**Additional file 10: Dataset S2.**  The detailed information of WGCNA analyses.

**Additional file 11: Fig. S5.** The transcriptional networks of MSBRM_0367, MSBRM_0968, MSBRM_0203, and MSBRM_2051. (a) The transcriptional network of MSBRM_0367. (b) The transcriptional network of MSBRM_0968. (c) The transcriptional network of MSBRM_0203. (d) The transcriptional network of MSBRM_2051. Nodes in the network represent genes, and gray lines link genes with the top 50 pairwise TOM values; thicker lines indicate higher TOM values. The background colors of nodes indicate which module they belong to (the detailed information of nodes is listed in the Additional file 10: Dataset S2).

**Additional file 12: Text S1.** The supplementary notes about materials, methods and WGCNA analyses.

**Additional file 13: Dataset S3.** The detailed information of proteomic analysis.

**Additional file 14: Fig. S6.** Flow chart of setting up different levels of acetate stress.

**Additional file 15: Table S5.** Acetate concentration in full-scale anaerobic digestors.

## Data Availability

The sequence datasets generated during the current work are available in the NCBI Sequence Read Archive and iProX, accession numbers provided in the text. The datasets supporting the conclusions of this article are uploaded as additional materials.

## Abbreviations

AD: anaerobic digestion
AtpF: V-type ATP synthase subunit F
DEGs: different expressed genes
DEPs: different expressed proteins
Dsb: DNA double-strand break
DppB: dipeptide transport system permease protein
DppF: dipeptide transport system ATP-binding protein
Ech: energy-conserving hydrogenase
Fts: cell division proteins
FrhA: coenzyme F420 hydrogenase subunit alpha
FrhB: coenzyme F420 hydrogenase subunit beta
FrhG: coenzyme F420 hydrogenase subunit gamma
Gs: glutamine synthetase
Gdh: glutamate dehydrogenase
Hdr: heterodisulfide reductase
Hsp: heat shock protein
lig1: ATP-dependent DNA ligase
Mtr: tetrahydromethanopterin S-methyltransferase
Nif: nitrogenase molybdenum–iron protein
OppA: periplasmic oligopeptide-binding protein
OppC: oligopeptide transport system permease protein
OppD: oligopeptide transport system ATP-binding protein
Pst: phosphate transport system
Pro: glycine betaine transport system
QS: quorum sensing system
Vhx: methanophenazine-reducing hydrogenase
Vht: methanophenazine-reducing hydrogenase
Vnf: nitrogenase vanadium–iron protein

## References

[1] Lü F, Hua Z, Shao L, He P (2018). Loop bioenergy production and carbon sequestration of polymeric waste by integrating biochemical and thermochemical conversion processes: a conceptual framework and recent advances. Renew Energy.

[2] Horn MA, Matthies C, Kusel K, Schramm A, Drake HL (2003). Hydrogenotrophic methanogenesis by moderately acid-tolerant methanogens of a methane-emitting acidic peat. Appl Environ Microbiol.

[3] Lins P, Reitschuler C, Illmer P (2014). *Methanosarcina* spp., the key to relieve the start-up of a thermophilic anaerobic digestion suffering from high acetic acid loads. Bioresour Technol..

[4] Lins P, Reitschuler C, Illmer P (2012). Development and evaluation of inocula combating high acetate concentrations during the start-up of an anaerobic digestion. Bioresour Technol.

[5] Lu F, Hao L, Guan D, Qi Y, Shao L, He P (2013). Synergetic stress of acids and ammonium on the shift in the methanogenic pathways during thermophilic anaerobic digestion of organics. Water Res.

[6] Angelidaki I, Ahring BK (1993). Thermophilic anaerobic-digestion of livestock waste—the effect of ammonia. Appl Microbiol Biotechnol.

[7] Luo G, Fotidis IA, Angelidaki I (2016). Comparative analysis of taxonomic, functional, and metabolic patterns of microbiomes from 14 full-scale biogas reactors by metagenomic sequencing and radioisotopic analysis. Biotechnol Biofuels.

[8] Tsapekos P, Kougias PG, Treu L, Campanaro S, Angelidaki I (2017). Process performance and comparative metagenomic analysis during co-digestion of manure and lignocellulosic biomass for biogas production. Appl Energy.

[9] Kurade MB, Saha S, Salama ES, Patil SM, Govindwar SP, Jeon BH (2019). Acetoclastic methanogenesis led by *Methanosarcina* in anaerobic co-digestion of fats, oil and grease for enhanced production of methane. Bioresour Technol.

[10] Luo C, Lu F, Shao L, He P (2015). Application of eco-compatible biochar in anaerobic digestion to relieve acid stress and promote the selective colonization of functional microbes. Water Res.

[11] De Vrieze J, Hennebel T, Boon N, Verstraete W (2012). *Methanosarcina*: the rediscovered methanogen for heavy duty biomethanation. Bioresour Technol.

[12] Fukuzaki S, Nishio N, Nagai S (1990). Kinetics of the methanogenic fermentation of acetate. Appl Environ Microbiol.

[13] Hao L, Bize A, Conteau D, Chapleur O, Courtois S, Kroff P (2016). New insights into the key microbial phylotypes of anaerobic sludge digesters under different operational conditions. Water Res.

[14] Maestrojuan GM, Boone DR (1991). Characterization of *Methanosarcina barkeri* MS^T^ and 227, *Methanosarcina mazei* S-6^T^, and *Methanosarcina vacuolata* Z-76I^T^. Int J Syst Bacteriol.

[15] Galagan JE, Nusbaum C, Roy A, Endrizzi MG, Macdonald P, FitzHugh W (2002). The genome of *M. acetivorans* reveals extensive metabolic and physiological diversity. Genome Res..

[16] Welander PV, Metcalf WW (2005). Loss of the *mtr* operon in *Methanosarcina* blocks growth on methanol, but not methanogenesis, and reveals an unknown methanogenic pathway. Proc Natl Acad Sci USA.

[17] Qu X, Mazeas L, Vavilin VA, Epissard J, Lemunier M, Mouchel JM (2009). Combined monitoring of changes in δ^13^CH_4_ and archaeal community structure during mesophilic methanization of municipal solid waste. FEMS Microbiol Ecol.

[18] Hao L-P, Lü F, Li L, Shao L-M, He P-J (2012). Shift of pathways during initiation of thermophilic methanogenesis at different initial pH. Bioresour Technol.

[19] Hao L, Lü F, Li L, Wu Q, Shao L, He P (2013). Self-adaption of methane-producing communities to pH disturbance at different acetate concentrations by shifting pathways and population interaction. Bioresour Technol.

[20] Kulkarni G, Kridelbaugh DM, Guss AM, Metcalf WW (2009). Hydrogen is a preferred intermediate in the energy-conserving electron transport chain of *Methanosarcina barkeri*. Proc Natl Acad Sci USA.

[21] Kulkarni G, Mand TD, Metcalf WW (2018). Energy conservation via hydrogen cycling in the methanogenic archaeon *Methanosarcina barkeri*. MBio..

[22] Lovley DR (2018). The hydrogen economy of *Methanosarcina barkeri*: life in the fast lane. J Bacteriol..

[23] Thauer RK, Kaster AK, Seedorf H, Buckel W, Hedderich R (2008). Methanogenic archaea: ecologically relevant differences in energy conservation. Nat Rev Microbiol.

[24] Hao L, McIlroy SJ, Kirkegaard RH, Karst SM, Fernando WEY, Aslan H (2018). Novel prosthecate bacteria from the candidate phylum Acetothermia. ISME J.

[25] Treu L, Campanaro S, Kougias PG, Zhu X, Angelidaki I (2016). Untangling the effect of fatty acid addition at species level revealed different transcriptional responses of the biogas microbial community members. Environ Sci Technol.

[26] Wirth R, Kovacs E, Maroti G, Bagi Z, Rakhely G, Kovacs KL (2012). Characterization of a biogas-producing microbial community by short-read next generation DNA sequencing. Biotechnol Biofuels.

[27] Li P, Fu X, Chen M, Zhang L, Li S (2019). Proteomic profiling and integrated analysis with transcriptomic data bring new insights in the stress responses of *Kluyveromyces marxianus* after an arrest during high-temperature ethanol fermentation. Biotechnol Biofuels.

[28] Zhu H, Ren X, Wang J, Song Z, Shi M, Qiao J (2013). Integrated OMICS guided engineering of biofuel butanol-tolerance in photosynthetic *Synechocystis* sp. PCC 6803. Biotechnol Biofuels..

[29] He M-X, Wu B, Shui Z-X, Hu Q-C, Wang W-G, Tan F-R (2012). Transcriptome profiling of *Zymomonas mobilis* under ethanol stress. Biotechnol Biofuels..

[30] Jetten MSM, Stams AJM, Zehnder AJB (1992). Methanogenesis from acetate—a comparison of the acetate metabolism in *Methanothrix soehngenii* and *Methanosarcina* spp. FEMS Microbiol Lett.

[31] Forgac M (2007). Vacuolar ATPases: rotary proton pumps in physiology and pathophysiology. Nat Rev Mol Cell Biol.

[32] Lovley DR, Ferry JG (1985). Production and consumption of H_2_ during growth of *Methanosarcina* spp. on acetate. Appl Environ Microbiol..

[33] Schmidt KL, Peterson ND, Kustusch RJ, Wissel MC, Graham B, Phillips GJ (2004). A Predicted ABC transporter, FtsEX, is needed for cell division in *Escherichia coli*. J Bacteriol.

[34] Hao L, Lu F, Mazeas L, Desmond-Le Quemener E, Madigou C, Guenne A (2015). Stable isotope probing of acetate fed anaerobic batch incubations shows a partial resistance of acetoclastic methanogenesis catalyzed by *Methanosarcina* to sudden increase of ammonia level. Water Res.

[35] Guss AM, Mukhopadhyay B, Zhang JK, Metcalf WW (2005). Genetic analysis of mch mutants in two *Methanosarcina* species demonstrates multiple roles for the methanopterin-dependent C-1 oxidation/reduction pathway and differences in H_2_ metabolism between closely related species. Mol Microbiol..

[36] Mand TD, Kulkarni G, Metcalf WW (2018). Genetic, biochemical, and molecular characterization of *Methanosarcina barkeri* mutants lacking three distinct classes of hydrogenase. J Bacteriol..

[37] Rees DC, Johnson E, Lewinson O (2009). ABC transporters: the power to change. Nat Rev Mol Cell Biol.

[38] Sandkvist M (2001). Type II secretion and pathogenesis. Infect Immun.

[39] Lane N, Martin W (2010). The energetics of genome complexity. Nature.

[40] Kato S, Kosaka T, Watanabe K (2008). Comparative transcriptome analysis of responses of *Methanothermobacter thermautotrophicus* to different environmental stimuli. Environ Microbiol.

[41] Rao NN, Torriani A (2010). Molecular aspects of phosphate transport in *Escherichia coli*. Mol Microbiol..

[42] Zhang L, Jahng D (2012). Long-term anaerobic digestion of food waste stabilized by trace elements. Waste Manag.

[43] Romero-Güiza M, Vila J, Mata-Alvarez J, Chimenos J, Astals S (2016). The role of additives on anaerobic digestion: a review. Renew Sustain Energy Rev.

[44] Litwin CM, Calderwood SB (1993). Role of iron in regulation of virulence genes. Clin Microbiol Rev.

[45] Tanaka KJ, Song S, Mason K, Pinkett HW (2018). Selective substrate uptake: the role of ATP-binding cassette (ABC) importers in pathogenesis. Biochim Biophys Acta Biomembr.

[46] Liu Y, Zhang Y, Quan X, Chen S, Zhao H (2011). Applying an electric field in a built-in zero valent iron–anaerobic reactor for enhancement of sludge granulation. Water Res.

[47] Rotaru A-E, Calabrese F, Stryhanyuk H, Musat F, Shrestha PM, Weber HS (2018). Conductive particles enable syntrophic acetate oxidation between *Geobacter* and *Methanosarcina* from coastal sediments. mBio.

[48] Esther B-O, Nik ABNM, Bert P (2006). A sensor for intracellular ionic strength. Proc Natl Acad Sci USA.

[49] Yan Z, Ferry JG (2018). Electron bifurcation and confurcation in methanogenesis and reverse methanogenesis. Front Microbiol..

[50] Rotaru A-E, Shrestha PM, Liu F, Markovaite B, Chen S, Nevin KP (2014). Direct interspecies electron transfer between *Geobacter metallireducens* and *Methanosarcina barkeri*. Appl Environ Microbiol.

[51] Yan Z, Wang M, Ferry JG (2017). A ferredoxin- and F_420_H_2_-dependent, electron-bifurcating, heterodisulfide reductase with homologs in the domains *Bacteria* and *Archaea*. mBio..

[52] Holmes DE, Rotaru A-E, Ueki T, Shrestha PM, Ferry JG, Lovley DR (2018). Electron and proton flux for carbon dioxide reduction in *Methanosarcina barkeri* during direct interspecies electron transfer. Front Microbiol..

[53] Harms U, Thauer RK (1997). Identification of the active site histidine in the corrinoid protein MtrA of the energy-conserving methyltransferase complex from *Methanobacterium thermoautotrophicum*. Eur J Biochem.

[54] Guss AM, Kulkarni G, Metcalf WW (2009). Differences in hydrogenase gene expression between *Methanosarcina acetivorans* and *Methanosarcina barkeri*. J Bacteriol.

[55] Raymond J, Siefert JL, Staples CR, Blankenship RE (2004). The natural history of nitrogen fixation. Mol Biol Evol.

[56] Zehr JP, Jenkins BD, Short SM, Steward GF (2010). Nitrogenase gene diversity and microbial community structure: a cross-system comparison. Environ Microbiol.

[57] Ja L (2000). Nitrogen fixation in methanogens: the archaeal perspective. Curr Issues Mol Biol..

[58] Mehta MP, Baross JA (2006). Nitrogen fixation at 92 °C by a hydrothermal vent archaeon. Science.

[59] Chien YT, Auerbuch V, Brabban AD, Zinder SH (2000). Analysis of genes encoding an alternative nitrogenase in the archaeon *Methanosarcina barkeri* 227. J Bacteriol.

[60] Murray PA, Zinder SH (1984). Nitrogen fixation by a methanogenic archaebacterium. Nature.

[61] Brul S, Coote P (1999). Preservative agents in foods: mode of action and microbial resistance mechanisms. Int J Food Microbiol.

[62] Menzel U, Gottschalk G (1985). The internal pH of *Acetobacterium wieringae* and *Acetobacter aceti* during growth and production of acetic acid. Arch Microbiol.

[63] Cabello P, Roldan MD, Moreno-Vivian C (2004). Nitrate reduction and the nitrogen cycle in archaea. Microbiology.

[64] Smith DP, Thrash JC, Nicora CD, Lipton MS, Burnum-Johnson KE, Carini P (2013). Proteomic and transcriptomic analyses of “*Candidatus* Pelagibacter ubique” describe the first PII-independent response to nitrogen limitation in a free-living Alphaproteobacterium. mBio.

[65] Maier T, Guell M, Serrano L (2009). Correlation of mRNA and protein in complex biological samples. FEBS Lett.

[66] Taniguchi Y, Choi PJ, Li GW, Chen H, Babu M, Hearn J (2010). Quantifying *E. coli* proteome and transcriptome with single-molecule sensitivity in single cells. Science..

[67] Jager D, Sharma CM, Thomsen J, Ehlers C, Vogel J, Schmitz RA (2009). Deep sequencing analysis of the *Methanosarcina mazei* Gö1 transcriptome in response to nitrogen availability. Proc Natl Acad Sci USA..

[68] Min H, Zinder SH (1989). Kinetics of acetate utilization by two thermophilic acetotrophic methanogens: *Methanosarcina* sp. strain CALS-1 and *Methanothrix* sp. strain CALS-1. Appl Environ Microbiol..

[69] Westermann P, Ahring BK, Mah RA (1989). Threshold acetate concentrations for acetate catabolism by aceticlastic methanogenic bacteria. Appl Environ Microbiol.

[70] Zhou Y, Chen Y, Chen S, Gu J (2018). fastp: an ultra-fast all-in-one FASTQ preprocessor. Bioinformatics.

[71] Kim D, Langmead B, Salzberg SL (2015). HISAT: a fast spliced aligner with low memory requirements. Nat Methods.

[72] Pertea M, Pertea GM, Antonescu CM, Chang TC, Mendell JT, Salzberg SL (2015). StringTie enables improved reconstruction of a transcriptome from RNA-seq reads. Nat Biotechnol.

[73] Robinson MD, McCarthy DJ, Smyth GK (2010). edgeR: a bioconductor package for differential expression analysis of digital gene expression data. Bioinformatics.

[74] Yu G, Wang LG, Han Y, He QY (2012). clusterProfiler: an R package for comparing biological themes among gene clusters. OMICS.

[75] Langfelder P, Horvath S (2008). WGCNA: an R package for weighted correlation network analysis. BMC Bioinform.

[76] Krogh A, Larsson B, Von Heijne G, Sonnhammer EL (2001). Predicting transmembrane protein topology with a hidden Markov model: application to complete genomes. J Mol Biol.

[77] Yu NY, Wagner JR, Laird MR, Melli G, Rey S, Lo R (2010). PSORTb 3.0: improved protein subcellular localization prediction with refined localization subcategories and predictive capabilities for all prokaryotes. Bioinformatics..

[78] Petersen TN, Brunak S, von Heijne G, Nielsen H (2011). SignalP 4.0: discriminating signal peptides from transmembrane regions. Nat Methods..

