# Supplementary material for: Responses of Methanosarcina barkeri to acetate stress
Source: Biotechnol Biofuels. 2019 Dec 16;12:289. doi: 10.1186/s13068-019-1630-5 (PMC6913021; doi:10.1186/s13068-019-1630-5)
Supplement: Supplementary file 1 — Additional file 1: Table S1. The concentration of total acetate and free acetic acid in each group. [file 13068_2019_1630_MOESM1_ESM.docx]

**Table S1. The concentration of total acetate and free acetic acid in each group**

| Days | Sample*^a^* | pH | Total acetate*^b^* (mM) | Free acetic acid*^b,c^* (mM) |
| --- | --- | --- | --- | --- |
| 0 | 10-group-1 | 6.160 | 11.89 | 0.45 |
|  | 10-group-2 | 6.290 | 11.26 | 0.32 |
|  | 10-group-3 | 6.353 | 11.34 | 0.28 |
|  | 25-group-1 | 6.224 | 28.07 | 0.93 |
|  | 25-group-2 | 6.231 | 25.23 | 0.82 |
|  | 25-group-3 | 6.316 | 24.96 | 0.67 |
|  | 50-group-1 | 6.273 | 40.14 | 1.19 |
|  | 50-group-2 | 6.266 | 46.97 | 1.42 |
|  | 50-group-3 | 6.348 | 47.91 | 1.20 |
| 3 | 10-group-1 | 5.915 | 13.41 | 0.88 |
|  | 10-group-2 | 6.025 | 10.60 | 0.55 |
|  | 10-group-3 | 6.362 | 8.47 | 0.21 |
|  | 25-group-1 | 6.008 | 24.71 | 1.32 |
|  | 25-group-2 | 5.986 | 22.91 | 1.28 |
|  | 25-group-3 | 6.123 | 20.25 | 0.84 |
|  | 50-group-1 | 6.260 | 54.39 | 1.67 |
|  | 50-group-2 | 6.064 | 46.55 | 2.20 |
|  | 50-group-3 | 6.305 | 46.14 | 1.28 |

*^a^*The biological triplications were measured.

*^b^*There are significant differences among three groups (*P* < 0.05).

*^c^*The correlation between the concentration of free acetic acid and total acetate is shown in Eq. (1).

$C_{free acetic acid}=\frac{C_{\mathrm{total}}\times C_{H^{+}}}{Ka+C_{H^{+}}}$ Eq. (1)

Where $C_{free acetic acid}$ is the free acetic acid concentration (M), $C_{\mathrm{total}}$ is the total acetate concentration (M), $C_{H^{+}}$ is the proton concentration (M), and Ka is the dissociation constant of acetate, which is about $Ka=1.74\times{10}^{-5}$.
