# Supplementary material for: Responses of Methanosarcina barkeri to acetate stress
Source: Biotechnol Biofuels. 2019 Dec 16;12:289. doi: 10.1186/s13068-019-1630-5 (PMC6913021; doi:10.1186/s13068-019-1630-5)
Supplement: Supplementary file 3 — Additional file 3: Table S2. The parameter values of each fitted curve. [file 13068_2019_1630_MOESM3_ESM.docx]

**Table S2. The parameter values of each fitted curve.**

| Parameter | Group | Boltzmann function*^a^* | | | | |
| --- | --- | --- | --- | --- | --- | --- |
|  |  | A_1_ | A_2_ | x_0_ | dx | R^2^ |
| Total CH_3_COO^-^ | 10-group | 11.57 mM | 0.83 mM | 20.17 d | 2.52 d | 0.97 |
|  | 25-group | 25.25 mM | 1.57 mM | 19.75 d | 3.42 d | 0.99 |
|  | 50-group | 50.72 mM | 2.28 mM | 30.70 d | 6.75 d | 0.97 |
| Cumulated CH_4_ | 10-group | 0.16 mmol | 0.91 mmol | 20.46 d | 2.29 d | 0.94 |
|  | 25-group | 0.13 mmol | 1.85 mmol | 16.21 d | 1.76 d | 0.98 |
|  | 50-group | 0.22 mmol | 3.40 mmol | 24.32 d | 5.54 d | 0.94 |
| pH | 10-group | 6.15 | 6.89 | 18.02 d | 3.95 d | 0.96 |
|  | 25-group | 6.05 | 7.21 | 14.81 d | 5.36 d | 0.97 |
|  | 50-group | 6.10 | 7.57 | 21.58 d | 8.46 d | 0.99 |
| OD600 | 10-group | 2.25 | 0.50 | 3.74 d | 21.75 d | 0.99 |
|  | 25-group | 2.24 | 0.45 | 6.37 d | 22.32 d | 0.99 |
|  | 50-group | 2.03 | 0.86 | 1.28 d | 15.09 d | 0.98 |

*^a^* The form of Boltzmann function is as follows:

$y = A_{2}+ \frac{A_{1}-A_{2}}{1+e^{\frac{x-x_{0}}{\mathrm{dx}}}}$ Eq. (1)

where A_1_ is the initial value, A_2_ is the terminal value, x_0_ is the value where y is equal to (A_1_ + A_2_)/2, and dx is the time constant.
