# Supplementary material for: Responses of Methanosarcina barkeri to acetate stress
Source: Biotechnol Biofuels. 2019 Dec 16;12:289. doi: 10.1186/s13068-019-1630-5 (PMC6913021; doi:10.1186/s13068-019-1630-5)
Supplement: Supplementary file 4 — Additional file 4: Table S3. Maximum and minimum rates of 10-, 25- and 50-group. [file 13068_2019_1630_MOESM4_ESM.docx]

**Table S3. Maximum and minimum rates of 10-, 25- and 50-group.**

| **Group** | **Type** | **OD600 descending rate**  **(d^-1^)** | **Acetate consumption rate**  **(mM/d)** | **CH_4_ yield rate**  **(mmol/d)** | **pH rising rate**  **(d^-1^)** |
| --- | --- | --- | --- | --- | --- |
| 10-group | maximum | 0.020 | 1.1 | 0.082 | 0.047 |
|  | minimum | 0.0043 | 8.0×10^-8^ | 1.2×10^-9^ | 1.3×10^-6^ |
| 25-group | maximum | 0.020 | 1.7 | 0.24 | 0.054 |
|  | minimum | 0.0050 | 1.2×10^-5^ | 8.9×10^-13^ | 1.9×10^-5^ |
| 50-group | maximum | 0.019 | 1.8 | 0.14 | 0.043 |
|  | minimum | 0.0011 | 0.044 | 0.00037 | 0.0010 |
