# Supplementary figures and images for: Responses of Methanosarcina barkeri to acetate stress
Source: Biotechnol Biofuels. 2019 Dec 16;12:289. doi: 10.1186/s13068-019-1630-5 (PMC6913021; doi:10.1186/s13068-019-1630-5)

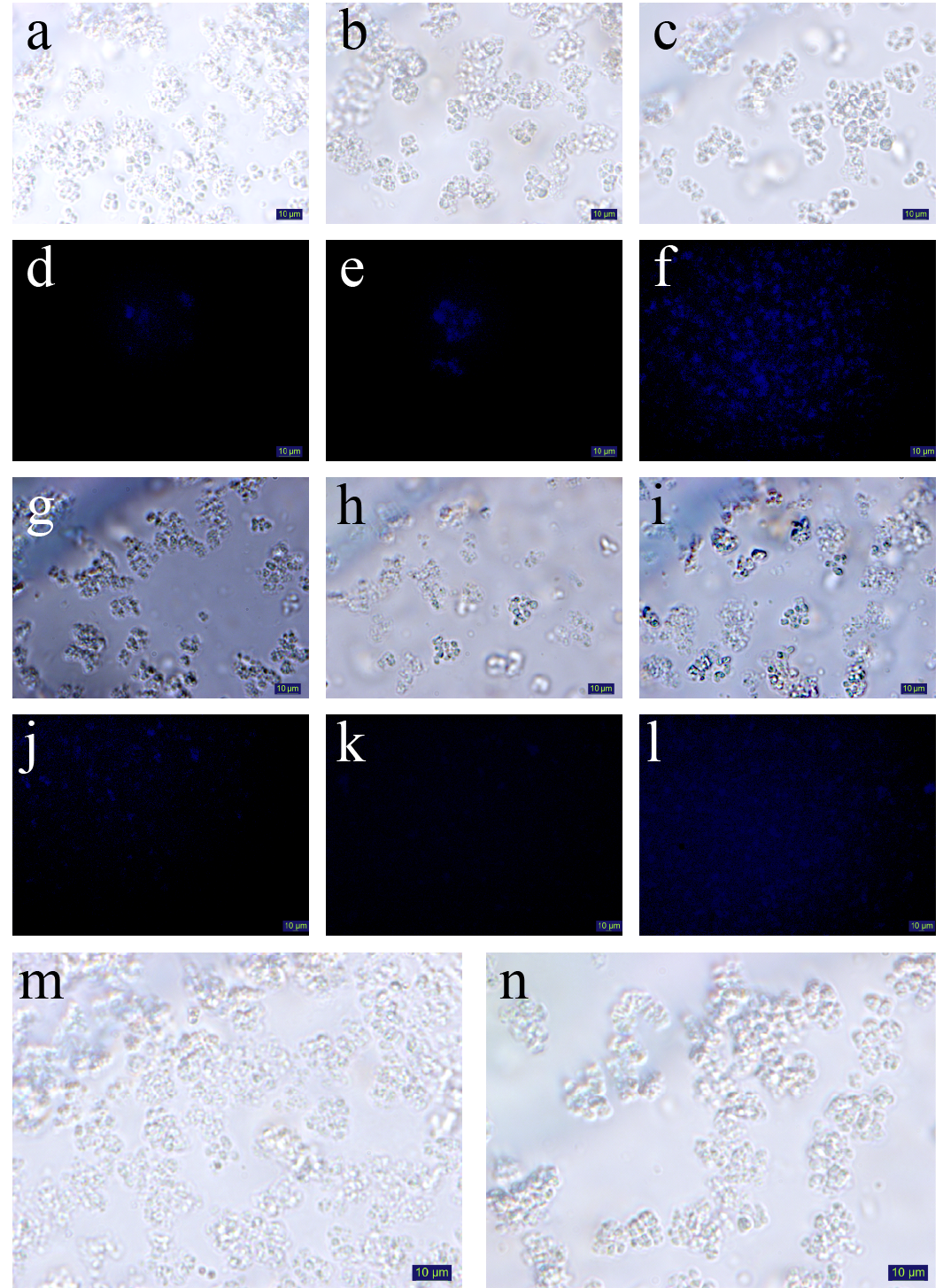

Supplement: Supplementary file 5 — Additional file 5: Fig. S2. The brightfield and epifluorescence micrographs of M. barkeri MS from different sampling points. (a), (d), and (m) 10-I; (b), (e), and (n) 25-I; (c) and (f) 50-I; (g) and (j) 10-T; (h) and (k) 25-T; (i) and (l) 50-T. [file 13068_2019_1630_MOESM5_ESM.tif]

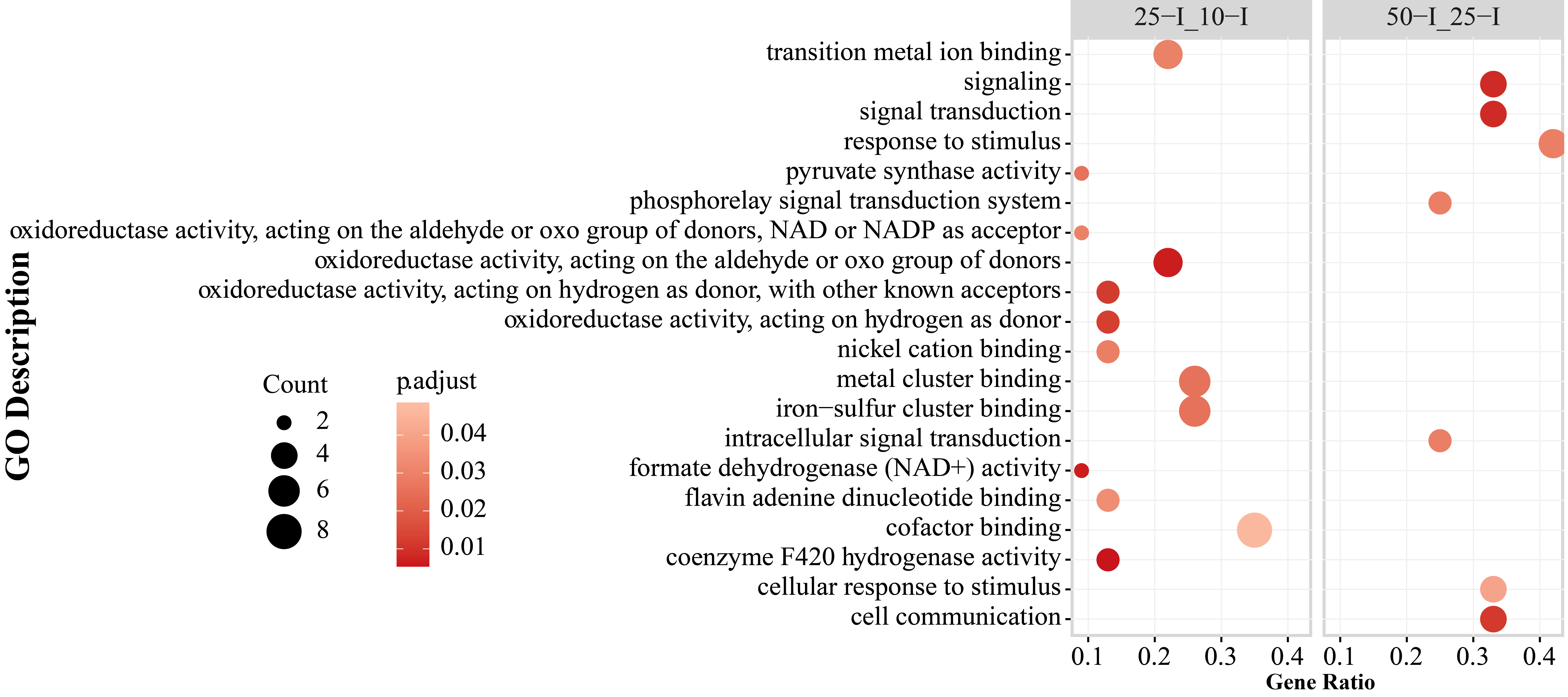

Supplement: Supplementary file 9 — Additional file 9: Fig. S4. GO enrichment analysis of downregulated DEGs in “25-I_10-I” and “50-I_25-I”. [file 13068_2019_1630_MOESM9_ESM.tif]

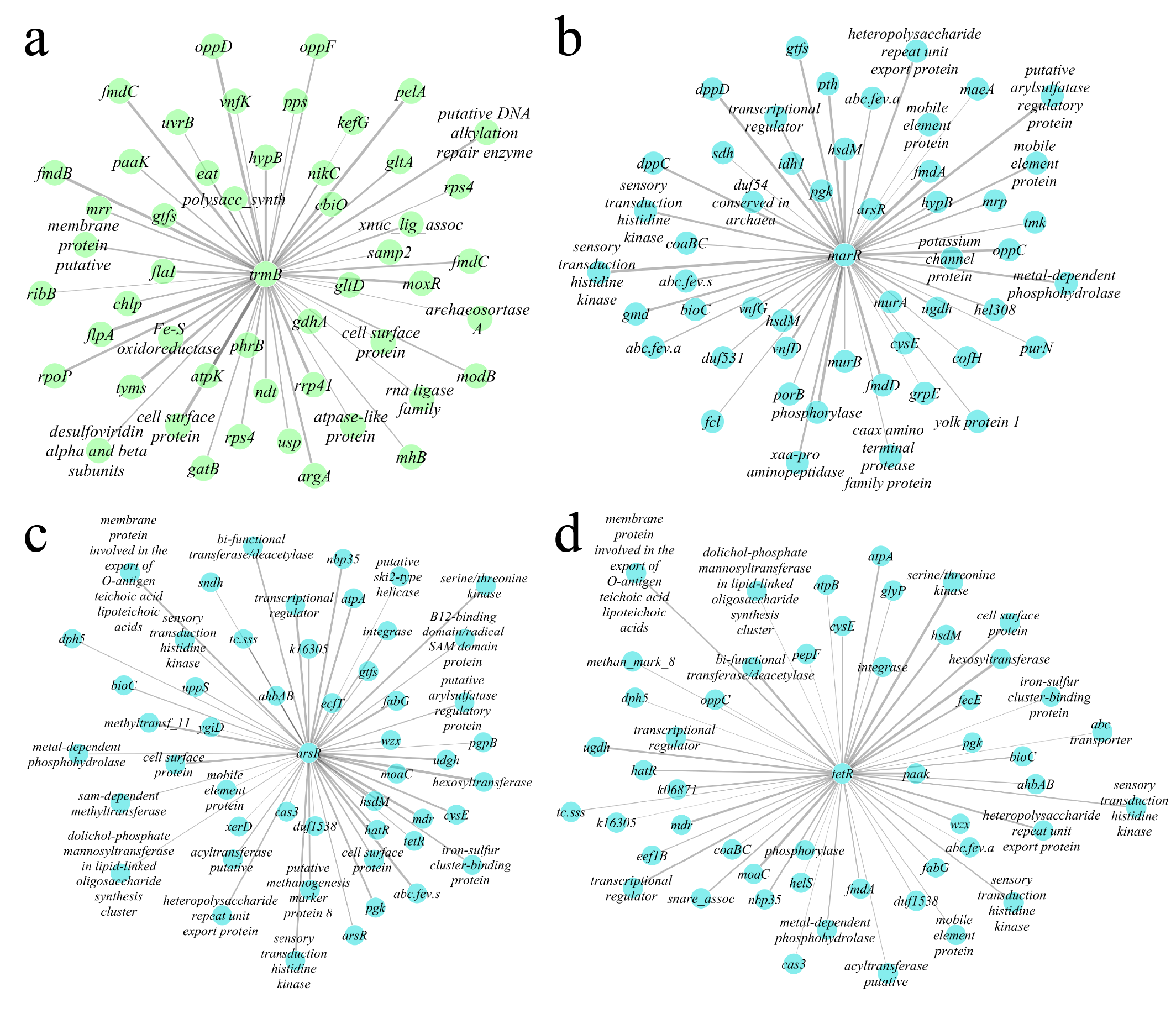

Supplement: Supplementary file 11 — Additional file 11: Fig. S5. The transcriptional networks of MSBRM_0367, MSBRM_0968, MSBRM_0203, and MSBRM_2051. (a) The transcriptional network of MSBRM_0367. (b) The transcriptional network of MSBRM_0968. (c) The transcriptional network of MSBRM_0203. (d) The transcriptional network of MSBRM_2051. Nodes in the network represent genes, and gray lines link genes with the top 50 pairwise TOM values; thicker lines indicate higher TOM values. The background colors of nodes indicate which module they belong to (the detailed information of nodes is listed in the Additional file 10: Dataset S2). [file 13068_2019_1630_MOESM11_ESM.tif]

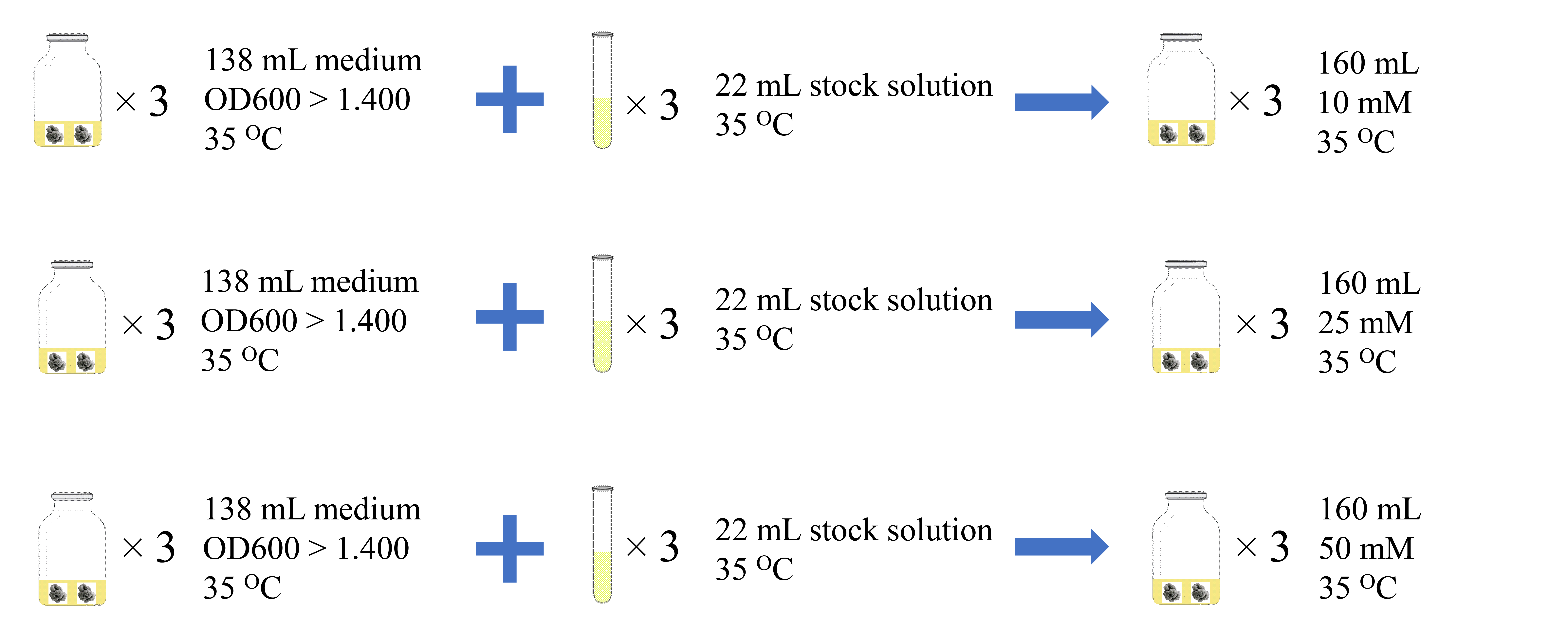

Supplement: Supplementary file 14 — Additional file 14: Fig. S6. Flow chart of setting up different levels of acetate stress. [file 13068_2019_1630_MOESM14_ESM.tif]
