# Supplementary material for: Responses of Methanosarcina barkeri to acetate stress
Source: Biotechnol Biofuels. 2019 Dec 16;12:289. doi: 10.1186/s13068-019-1630-5 (PMC6913021; doi:10.1186/s13068-019-1630-5)
Supplement: Supplementary file 6 — Additional file 6: Table S4. The transcriptomic sequencing data analysis. (a) The counts of filtered non-rRNA reads mapped to the reference genome. (b) Fold changes of differentially expressed genes from RNA-seq and RT-qPCR in different comparisons. [file 13068_2019_1630_MOESM6_ESM.docx]

**Table S4. The transcriptomic sequencing data analysis.** a) The counts of filtered non-rRNA reads mapped to the reference genome. b) Fold changes of differentially expressed genes from RNA-seq and RT-qPCR in different comparison.

a)

| Sample name | Read counts |
| --- | --- |
| 10-I | 23692410 |
| 25-I | 18436257 |
| 50-I | 20628816 |
| 10-T | 18571582 |
| 25-T | 14024581 |
| 50-T | 13431348 |

b)

| Locus_tag | Annotation*^a^* | Comparison | RNA-seq | RT-qPCR |
| --- | --- | --- | --- | --- |
| MSBRM_1085 | CO dehydrogenase/acetyl-CoA synthase subunit delta | “25-T_10-I” | 4.67*^b^* | 1.88 |
|  |  | “50-T_50-I” | 2.67*^b^* | 2.71*^b^* |
| MSBRM_0271 | Transcriptional regulator | “50-I_10-I” | 0.79 | 0.51 |
|  |  | “10-T_10-I” | 2.93*^b^* | 2.35 |
|  |  | “25-T_25-I” | 2.26*^b^* | 1.76*^b^* |
| MSBRM_3457 | Polyphosphate kinase | “50-I_10-I” | 0.70 | 0.85 |
|  |  | “10-T_10-I” | 2.28*^b^* | 1.61 |
| MSBRM_0391 | Formylmethanofuran dehydrogenase subunit A | “25-I_10-I” | 0.69 | 0.8 |
| MSBRM_1427 | Response regulator receiver | “50-T_50-I” | 6.61*^b^* | 3.39 |
| MSBRM_1541 | Methylthiol:coenzyme M methyltransferase corrinoid protein | “25-T_25-I” | 2.02*^b^* | 2.11 |

*^a^*Annotation according to Ensembl Genomes database.

*^b^*Value with statistical significance (*P*<0.05).
