# Supplementary material for: Responses of Methanosarcina barkeri to acetate stress
Source: Biotechnol Biofuels. 2019 Dec 16;12:289. doi: 10.1186/s13068-019-1630-5 (PMC6913021; doi:10.1186/s13068-019-1630-5)
Supplement: Supplementary file 12 — Additional file 12: Text S1. The supplementary notes about materials, methods and WGCNA analyses. [file 13068_2019_1630_MOESM12_ESM.docx]

**SI Results about WGCNA**

WGCNA identified 13 modules of genes and the minimum module size was 32 transcripts. These modules were distinguished by colors (**Dataset S2**). According to the selection criteria (correlation coefficient > |0.9| and p.value < 0.05), 80 and 59 genes (labeled as “P_Ac” and “P_pH” groups, respectively) were positively correlated with acetate consumption rates and pH values, respectively, of which 35 genes were identical (**Dataset S2**). In contrast, 100 and 115 genes (labeled as “N_Ac” and “N_pH” groups, respectively) were negatively correlated with acetate consumption rates and pH values, respectively, of which 56 genes were identical (**Dataset S2**). The high proportion of the identical genes indicated that acetate consumption rates were correlated with pH values, which was consistent with the fact that cells could only perform physiological activities in suitable pH environments. The genes having negative correlations with acetate consumption rates and pH values might play essential roles in the initial stages of acetate stress. 6 and 144 genes (labeled as “P_CH_4_” and “P_OD” groups, respectively) were positively correlated with CH_4_ yield rates and OD600 values, respectively (**Dataset S2**).

The genes encoding stress proteins and transcriptional regulators in each group were selected for the further analyses of connectivity with other genes. Because hypothetical proteins lacked clear annotations, these proteins were not considered in networks. Gene MSBRM_0367, which encodes a transcriptional regulator, was assigned to both of the “N_Ac” and “N_pH” group. This gene might be essential in resisting acetate stress. Gene MSBRM_0367 was associated with the genes participating in nitrogen metabolism, quorum sensing, methane metabolism and other processes (**Dataset S2 & Fig. S5a**). In the “N_Ac” and “P_OD” groups, gene MSBRM_0968 was associated with the genes participating in nitrogen metabolism, quorum sensing and other processes (**Dataset S2 & Fig. S5b**). It indicated that gene MSBRM_0968 would be downregulated with OD600 values decreased. In addition to gene MSBRM_0367, genes MSBRM_0203 and MSBRM_2051 were also assigned to the “N_pH” group. These two genes were associated with the genes participating in energy synthesis, transcriptional regulation, sensory transduction, iron uptake and other processes. It indicated that these processes were essential for resisting acetate stress in the initial stages (**Dataset S2 & Fig. S5c & d**).

**SI MATERIALS AND METHODS**

**Pure strain and stress conditions**

*Methanosarcina barkeri* MS (DSM 800), which was purchased from Deutsche Sammlung von Mikroorganismen und Zellkulturen (DSMZ), was preincubated in 250 mL sealed serum bottles with a 138 mL medium volume at 35 °C. The medium was prepared according to the instructions provided by DSMZ with slight modifications (**Table 1-3**).

**Table 1.** **The components in culture medium.**

| Components | Concentration in distilled water |
| --- | --- |
| K_2_HPO_4_ | 0.35 mg/mL |
| KH_2_PO_4_ | 0.23 mg/mL |
| NH_4_Cl | 0.50 mg/mL |
| MgSO_4_ **×** 7 H_2_O | 0.50 mg/mL |
| CaCl_2_ **×** 2 H_2_O | 0.25 mg/mL |
| NaCl | 2.25 mg/mL |
| FeSO_4_ **×** 7 H_2_O solution (0.1% w/v in 0.1 H_2_SO_4_) | 2.00 μL/mL |
| Trace element solution SL-10 (see **Table 2**) | 1.00 μL/mL |
| Yeast extract (OXOID) | 2.00 mg/mL |
| Casitone (BD BBL) | 2.00 mg/mL |
| Na-resazurin solution (0.1% w/v) | 0.50 μL/mL |
| NaHCO_3_ | 0.85 mg/mL |
| Vitamin solution (**see Table 3**) | 10.00 μL/mL |
| Methanol | 10.00 μL/mL |
| L-Cysteine-HCl **×** H_2_O | 0.30 mg/mL |
| Na_2_S **×** 9 H_2_O | 0.30 mg/mL |

**Table 2. The trace element components in solution SL-10.**

| Components | Concentration in distilled water |
| --- | --- |
| HCl (25%; 7.7 M) | 10.00 μL/mL |
| FeCl_2_ **×** 4 H_2_O | 1.50 mg/mL |
| ZnCl_2_ | 70.00 μg/mL |
| MnCl_2_ **×** 4 H_2_O | 100.00 μg/mL |
| H_3_BO_3_ | 6.00 μg/mL |
| CoCl_2_ **×** 6 H_2_O | 190.00 μg/mL |
| CuCl_2_ **×** 2 H_2_O | 2.00 μg/mL |
| NiCl_2_ **×** 6 H_2_O | 24.00 μg/mL |
| Na_2_MoO_4_ **×** 2 H_2_O | 36.00 μg/mL |

**Table 3. The components in vitamin solution.**

| Components | Concentration in distilled water |
| --- | --- |
| Biotin | 2.00 μg/mL |
| Folic acid | 2.00 μg/mL |
| Pyridoxine-HCl | 10.00 μg/mL |
| Thiamine-HCl | 5.00 μg/mL |
| Riboflavin | 5.00 μg/mL |
| Nicotinic acid | 5.00 μg/mL |
| D-Ca-pantothenate | 5.00 μg/mL |
| Vitamin B_12_ | 0.10 μg/mL |
| p-Aminobenzoic acid | 5.00 μg/mL |
| Lipoic acid | 5.00 μg/mL |

The gas in the serum bottles was periodically released through sterile needles, and the gas volume was recorded. The stock solution contained CH_3_COONa, CH_3_COOK, CH_3_COOH, KCl, NaCl, and the above medium without methanol. When OD600 values reached 1.400, 22 mL stock solution was first filtered using sterile membrane filters (PVDF membrane, Merck Millipore Ltd, Carrigtwohill, Ireland), and then injected into the serum bottles through sterile syringes. After injection, there was a total of 160 mL culture solution in each serum bottle. These serum bottles were divided into three groups, named as 10-group, 25-group, and 50-group, in which the total acetate concentrations were set as 10, 25, and 50 mM, respectively (**Fig. S6**). And methanol concentrations decreased to levels below the detection limit. In engineered AD processes, acetate stress is similar to a shock. Therefore, substrate was switched from methanol to acetate to better reflect the responses of *M. barkeri*. The CH_3_COOH/CH_3_COO^-^ buffer solution maintained a suitable pH for *M. barkeri*. Sodium and potassium ion concentrations were adjusted to be the same in all three groups, through the addition of KCl and NaCl. It avoided the effects of inconsistent cation concentrations. All groups were conducted in triplicate and maintained at 35 ± 1 °C in the dark.

**Table 4. The components in stock solution.**

| Components | 10-group | 25-group | 50-group |
| --- | --- | --- | --- |
| Medium (**Table 1**) without methanol | 20.4 mL | 20.4 mL | 20.4 mL |
| CH_3_COOH/CH_3_COO^-^ buffer solution | 1.6 mL | 1.6 mL | 1.6 mL |
| KCl | 0.05 g | 0.03 g | − |
| NaCl | 0.34 g | 0.21 g | − |

**Table 5. The components in CH_3_COOH/CH_3_COO^-^ buffer solution.**

| Components | 10-group (50 mL) | 25-group (100 mL) | 50-group (100 mL) |
| --- | --- | --- | --- |
| CH_3_COOH | 25.7 μL | 128.6 μL | 257 μL |
| CH_3_COOK | 0.49 g | 2.46 g | 4.92 g |
| CH_3_COONa | 3.70 g | 18.52 g | 37.05 g |

**Sample measurements**

Sample frequency was determined by the results from pre-experiments (unpublished data). The gas pressure in the headspace of serum bottles was measured using a differential pressure meter (Testo 512, Testo SE & Co.KGaA, Lenzkirch, Germany). The volume (%) of CH_4_ was monitored using a gas chromatograph equipped with a flame ionization detector (Trace 1300, Thermo Fisher Scientific, Waltham, MA, USA). The OD600 value was measured using a spectrophotometer (Nanodrop 2000c, Thermo Scientific Co., Waltham, USA). The brightfield and epifluorescence micrographs were taken by a microscopy (DMI4000 B, Leica Microsystems CMS GmbH, Wetzlar, Germany). Approximately 8 mL of culture solution was collected into 2 mL centrifuge tubes (DNA or Protein Lobind Tube, PCR clean grade, RNase-free, Eppendorf AG, Hamburg, Germany) and centrifuged at 16000×*g* at 4 °C for 4 min (Sorvall ST-16R, Thermo Electron LED GmbH, Osterode, Germany). After centrifugation, the pH value of the supernatant was tested using a pH meter (PXSJ-216F, Shanghai Precision and Scientific Instrument Co., LTD, Shanghai, China). The acetate concentration of the supernatant was measured in triplicate using a gas chromatograph equipped with a flame ionization detector (Focus GC, Thermo Scientific Co., Waltham, USA). The trends of cumulative methane yields, OD600 values, pH values and acetate concentrations were fitted using the Boltzmann function, based on the average values at each sample point

**RNA extraction and sequencing**

After centrifugation, the pellets were flash frozen in liquid nitrogen and stored at -80 °C. RNA was extracted using TRIzol Reagent (Invitrogen, Carlsbad, California, USA) according to the manufacturer’s instructions. Quality assessment of total RNA was conducted using Nanodrop (Thermo Scientific Co., Waltham, USA) and Agilent 2100 (Agilent Technologies lnc., Santa Clara, USA). The RNA integrity number of each sample was equal to or greater than 7.0. High-quality RNA was used for RNA-seq and reverse transcription quantitative PCR (RT-qPCR) verification. Equal quantities of RNA from triplicate samples were pooled for RNA-seq. rRNAs were removed using the Ribo-Zero Magnetic kit (Illumina, San Diego, CA, USA). Then, the fragmented mRNAs were used to synthesize the first strand cDNA by random hexamers, which served as the template for the second cDNA. After adaptor ligation, six paired-end RNA-seq libraries were constructed using Illumina Truseq RNA sample Preparation Kit (Illumina, San Diego, CA, USA). The constructed libraries were purified with AMPure XP (Beckman coulter, IN, USA) and quantified using Agilent 2100 (Agilent Technologies lnc., Santa Clara, USA). Finally, purified paired-end libraries were sequenced using Illumina HiSeq 2500. The sequencings were conducted by Majorbio Bio-pharm Technology Co., Ltd, Shanghai, China.

**Transcriptomic analysis**

The raw reads were filtered to trim adapter sequences, remove the reads with nucleotides that contained more than 10% poly-N, and remove the low quality reads where nucleotides with Q-score ≤ 20 occupied more than 50% of the entire read using fastp (v0.12.3) [1]. rRNAs were removed from the trimmed clean reads, and the rest trimmed clean reads were aligned to the reference genome of *M. barkeri* MS from Ensembl Genomes using HISAT2 (v2.1.0; --no-spliced-alignment; --dta; other parameters set to default) [2,3]. The processed SAM files were sorted by chromosomal coordinates and converted to BAM files using Samtools (v1.9) [4]. Transcript assembly was performed based on the BAM files, and the generated GTF files were used to quantify read counts using Stringtie (v1.3.4) ([2,5]. Considering that no biological replicates was sequenced, differentially expressed genes (DEGs) were estimated by the R package edgeR (v3.22.3) [6]. The biological coefficient of variation (BCV) value was set as 0.1 to calculate the dispersion for the nonbiological replication of model organism, according to the user guide. Read counts were normalized by the “TMM” function in edgeR. DEGs were estimated using the “exactTest” function as in the previous study [7]. The selection criteria for DEGs was a fold change > 2 and false discovery rate < 0.05 (adjusted *P*-value corrected by Benjamini and Hochberg method) [8]. The GO and KEGG module enrichment analyses of DEGs were conducted using the R package clusterProfiler (v3.8.1) [9], and adjusted *P*-value < 0.05 (Benjamini and Hochberg methods) was used as the selection criteria.

To examine the relationships between gene expression patterns and phenotypic traits, the weighted correlation network analyses were conducted using the R package WGCNA (v1.66) [10]. The read counts were first normalized using a variance-stabilizing transformation by DESeq2 (v1.22.2) [11] according to previous studies [12]. Briefly, a soft-threshold of 9 was chosen for the “blockwiseModules” command to identify the transcriptional modules (minModuleSize = 30, mergeCutHeight = 0.25) [13]. The correlation coefficients between gene expression patterns and phenotypic traits were also calculated using WGCNA.

**qPCR verification**

The six DEGs participating in methane metabolism, transcription regulation and polyphosphate formation were verified by RT-qPCR analyses in triplicate using TB Green^TM^ *Premix Ex Taq*^TM^ II (Tli RNaseH Plus) (RR820A, Takara Bio Inc, Shiga, Japan) on a StepOnePlus^TM^ Real-time PCR system (Thermo Fisher, Waltham, MA, USA). The primers are listed in **Table 6.** The reactions were performed in a total of 10 μL, containing 5 μL 2×TB Green^TM^ *Premix Ex Taq*^TM^ II, 0.4 μL Primer F, 0.4 μL Primer R, 0.2 μL 50×ROX Reference Dye, 1 μL cDNA, and 3 μL ddH_2_O. The 16S rRNA of *M. barkeri* MS was selected as the reference to calibrate the expression levels of these DEGs. The PCR program consisted of an initial denaturation at 95 °C for 30 s, followed by 40 cycles of 5 s of denaturation at 95 °C and 30 s of annealing and elongation. The relative expression levels of DEGs were calculated using the comparative threshold (2^-△△Ct^) according to the previous study [14]. All of the qPCR verifications were conducted by Wcgene Biotech Co., Ltd, Shanghai, China.

**Table 6. Primers for RT-qPCR in present study.**

| Primer* | Sequence | Application |
| --- | --- | --- |
| MSBRM_3457_F | GATCGATCGCCCTGACCTTA | Polyphosphate kinase qPCR |
| MSBRM_3457_R | CTTCGGCAGATTCTTCCACG |  |
| MSBRM_0391_F | GGACATAGAAAAGGCTGCGG | Formylmethanofuran dehydrogenase subunit A qPCR |
| MSBRM_0391_R | TGCATGCAGGTGAAGAGGTA |  |
| MSBRM_1427_F | AGATTGCCTCCCGTCTTTCA | Response regulator receiver qPCR |
| MSBRM_1427_R | GTCTGCAAGCCCTGTATCCA |  |
| MSBRM_1085_F | CTCGGAAGCAGCTTTGATCC | CO dehydrogenase/acetyl-CoA synthase subunit delta qPCR |
| MSBRM_1085_R | GCTCGTTTGAACCCGCTATT |  |
| MSBRM_1541_F | CAAATGCTCTGGAACCCGAG | Methylthiol:coenzyme M methyltransferase corrinoid protein qPCR |
| MSBRM_1541_R | TAAAGTTCGTTGCGGGCTTC |  |
| MSBRM_0271_F | AGGCGGACGTGGTTACTATC | Transcriptional regulator qPCR |
| MSBRM_0271_R | ACAGGTTCAAGCCATTCTGC |  |

*Primers were designed by Primer (V5.0, Premier Biosoft, CA, USA).

**Isobaric tags for relative and absolute quantitation (iTRAQ) labeling and protein quantification**

The cell pellets collected from 10-I, 50-I, and 50-T were ground in liquid nitrogen, resuspended in a 10-fold volume of lysate (1% solution of sodium lauryl sulfate (SDS), 200 mM DTT, and 50 mM Tris-HCl, pH 8.8), homogenized by vortexing and lysed on ice. Each lysate was centrifuged at 4 °C (12000×*g*, 20 min). The supernatant was precipitated overnight in a 4-fold volume of precooling acetone and then centrifuged at 4 °C (12000×*g*, 20 min) again, and the supernatant was discarded. The precipitate was vortexed in 90% acetone for 10 s and then centrifuged at 4 °C (12000×*g*, 20 min). The precipitate obtained was treated again using the same method. Then the generated precipitate was lysed using an 8 M urea and 1% SDS solution and then centrifuged at 8 °C (12000×*g*, 20 min). The protein concentrations were determined to be 5.612, 4.291 and 3.449 mg/mL (10-I, 50-I and 50-T, respectively) by Bicinchoninic acid assay (Micro BCA, Thermo Fisher, Waltham, MA, USA), according to the manufacturer’s instructions. One hundred micrograms of each protein was suspended separately using lysate in a total volume of 90 μL. The suspended proteins were reacted with 10 mM Tris(2-carboxyethyl)phosphine (TCEP) at 37 °C for 60 min and then reacted with 40 mM iodoacetamide (Sigma) at room temperature for 40 min in the dark. Each sample was mixed with a six-fold volume of acetone at **–** 20 °C for 4 hours. After centrifugation (10000×*g*, 20 min), the precipitate was dissolved in 100 μL of 100 mM tetraethylammonium bromide (TEAB, Sigma). And then the proteins were digested using trypsin (Promega) at 37 °C overnight with an enzyme to protein ratio of 1:50. iTRAQ reagents (AB Sciex) were used to label the digested peptides following the manufacturer’s instructions. The 113-tag, 114-tag and 117-tag were used to indicate 10-I, 50-I, and 50-T, respectively. The three labeled samples were mixed and then fractionated by High pH Reverse-Phase (hpRP). The tryptic peptides labeled by iTRAQ were reconstituted in buffer A (an aqueous solution of 2% acetonitrile, pH 10 adjusted with NH_3_·H_2_O) and injected into ACQUITY UPLC BEH C18 Column (1.7 μm, 2.1 mm × 150 mm, Waters) using buffer A and buffer B (an aqueous solution of 80% acetonitrile, pH 10 adjusted with NH_3_·H_2_O). The liquid chromatogram process was run as described in **Table 7**. Twenty fractions were collected according to the peak shape and retention time. These fractions were pooled into a total of 10 fractions based on the absorbance of UV_214_. The 10 fractions were concentrated in a rotation vacuum concentrator (RVC 2-25, Martin Christ, Osterode am Harz, Germany) and reconstituted in 2% ACN with 0.1% formic acid, which was the buffer A for LC-MS/MS. The solution obtained was analyzed by a Q-Exactive mass spectrometer (Thermo-Fisher Scientific, San Jose, USA) with EASY-nLC 1200 system (Thermo-Fisher Scientific, San Jose, USA). Each fraction reconstituted was separated on a C18 reversed-phase LC column (75 μm × 25 cm, Thermo) using the gradient listed in **Table 8**. The column was re-equilibrated with buffer A for 20 min before the next fraction analysis. The inlet electrospray voltage of the Q-Exactive was set as 1.8 kV. Data-dependent acquisition (DDA) mode was selected as the instrument method for Q-Exactive. For each fraction analysis, a survey full-scan was executed in a mass range of *m/z* 350-1300 at a resolution of 70000. Then 20 most intensive precursors were selected for the second fragmentation using high energy collision dissociation (HCD) at a resolution of 17500. In the above cases, the dynamic exclusion of 18 s was selected to record one microscan. For MS/MS, a normalized collision energy of 30% was set. All raw data were collected using Thermo Xcalibur 4.0 software (Thermo-Fisher Scientific, San Jose, USA) and imported into Proteome Discoverer software (v2.1, Thermo-Fisher Scientific, Bremen, Germany). Protein identification and quantification were carried out by Sequest HT (Thermo-Fisher Scientific, Bremen, Germany) using the peptide sequences of *M. barkeri* MS from Ensembl Genomes. The parameters are listed in **Table 9**. Fold change > 1.2 or < 0.83 was selected to determine the upregulation and downregulation of proteins, respectively. All of the above processes were conducted by Majorbio Bio-pharm Technology Co., Ltd, Shanghai, China. The predictions of transmembrane helices in hypothetical proteins were conducted by TMHMM (v2.0) [15]. PSORTb (v3.0) was used to predict the proteins’ subcellular localization [16], and SignalP (v4.0) was used to predict the presence of signal peptides [17].

**Table 7.** **The gradient of buffer adopted for UPLC.**

| Time (min) | B (%) |
| --- | --- |
| 0 | 0 |
| 2 | 0 |
| 17 | 3.8 |
| 35 | 24 |
| 38 | 30 |
| 39 | 43 |
| 40 | 100 |
| 46 | 0 |
| 66 | Stop |

**Table 8.** **The gradient of buffer adopted for EASY-nLC.**

| Time (min) | B (%) |
| --- | --- |
| 0 | 0 |
| 1 | 5 |
| 41 | 23 |
| 58 | 48 |
| 59 | 100 |
| 65 | 100 |
| 66 | 0 |
| 70 | 0 |
| 90 | Stop |

**Table 9.** **Search parameters adopted for the ProteomeDiscoverer.**

| Item | Value |
| --- | --- |
| Software version | 2.1 |
| Protein Database | Methanosarcina_barkeri _ms.ASM97002v1.pep.fasta |
| Cys alkylation | Iodoacetamide |
| Dynamic Modification | Oxidation (M), Acetyl (Protein N-Terminus)，iTRAQ8plex (Y) |
| Static Modification | iTRAQ8plex (K), iTRAQ8plex (N-Terminus), Carbamidomethyl（C） |
| Enzyme Name | Trypsin (Full) |
| Max. Missed Cleavage Sites | 2 |
| Precursor Mass Tolerance | 10 ppm |
| Fragment Mass Tolerance | 0.05Da |
| Validation based on | q-value |

Note: The results were filter by peptide FDR ≤ 0.01.

**References:**

1. Zhou Y, Chen Y, Chen S, Gu J. fastp: an ultra-fast all-in-one FASTQ preprocessor. Bioinformatics. 2018;34(17):i884-i90.

2. Pertea M, Kim D, Pertea GM, Leek JT, Salzberg SL. Transcript-level expression analysis of RNA-seq experiments with HISAT, StringTie and Ballgown. Nat Protoc. 2016;11(9):1650-67.

3. Kim D, Langmead B, Salzberg SL. HISAT: a fast spliced aligner with low memory requirements. Nat Methods. 2015;12(4):357-60.

4. Li H, Handsaker B, Wysoker A, Fennell T, Ruan J, Homer N, et al. The Sequence Alignment/Map format and SAMtools. Bioinformatics. 2009;25(16):2078-9.

5. Pertea M, Pertea GM, Antonescu CM, Chang TC, Mendell JT, Salzberg SL. StringTie enables improved reconstruction of a transcriptome from RNA-seq reads. Nat Biotechnol. 2015;33(3):290-5.

6. Robinson MD, McCarthy DJ, Smyth GK. edgeR: a Bioconductor package for differential expression analysis of digital gene expression data. Bioinformatics. 2010;26(1):139-40.

7. Gong W, Browne J, Hall N, Schruth D, Paerl H, Marchetti A. Molecular insights into a dinoflagellate bloom. ISME J. 2017;11(2):439-52.

8. Benjamini Y, Hochberg Y. Controlling the False Discovery Rate: A Practical and Powerful Approach to Multiple Testing. Journal of the Royal Statistical Society Series B (Methodological). 1995;57(1):289-300.

9. Yu G, Wang LG, Han Y, He QY. clusterProfiler: an R package for comparing biological themes among gene clusters. OMICS. 2012;16(5):284-7.

10. Langfelder P, Horvath S. WGCNA: an R package for weighted correlation network analysis. BMC Bioinformatics. 2008;9(1):559.

11. Love MI, Huber W, Anders S. Moderated estimation of fold change and dispersion for RNA-seq data with DESeq2. Genome Biol. 2014;15(12):550.

12. Wilson ST, Aylward FO, Ribalet F, Barone B, Casey JR, Connell PE, et al. Coordinated regulation of growth, activity and transcription in natural populations of the unicellular nitrogen-fixing cyanobacterium Crocosphaera. Nat Microbiol. 2017;2(9):17118.

13. Harke MJ, Frischkorn KR, Haley ST, Aylward FO, Zehr JP, Dyhrman ST. Periodic and coordinated gene expression between a diazotroph and its diatom host. ISME J. 2019;13(1):118-31.

14. Livak KJ, Schmittgen TD. Analysis of Relative Gene Expression Data Using Real-Time Quantitative PCR and the 2^−ΔΔCT^ Method. Methods-A Companion To Methods in Enzymology. 2001;25(4):402-8.

15. Krogh A, Larsson B, Von Heijne G, Sonnhammer EL. Predicting transmembrane protein topology with a hidden Markov model: application to complete genomes. J Mol Biol. 2001;305(3):567-80.

16. Yu NY, Wagner JR, Laird MR, Melli G, Rey S, Lo R, et al. PSORTb 3.0: improved protein subcellular localization prediction with refined localization subcategories and predictive capabilities for all prokaryotes. Bioinformatics. 2010;26(13):1608-15.

17. Petersen TN, Brunak S, von Heijne G, Nielsen H. SignalP 4.0: discriminating signal peptides from transmembrane regions. Nat Methods. 2011;8(10):785.
