# Supplementary material for: Responses of Methanosarcina barkeri to acetate stress
Source: Biotechnol Biofuels. 2019 Dec 16;12:289. doi: 10.1186/s13068-019-1630-5 (PMC6913021; doi:10.1186/s13068-019-1630-5)
Supplement: Supplementary file 15 — Additional file 15: Table S5. Acetate concentration in full-scale anaerobic digestors. [file 13068_2019_1630_MOESM15_ESM.docx]

**Table S5. Acetate concentration in full-scale anaerobic digestors**

| Digestor Number | Volume capacity  （m³） | pH | Temperature  (℃) | Substrate | Acetate concentration (mM) | Free acetic acid (mM) | reference |
| --- | --- | --- | --- | --- | --- | --- | --- |
| 1 | 2,300 | - | 54 | Industrial food waste | < 16.67 | - | [1] |
| 2 | 12,600 | 7.37 | 34.6 | Primary sewage sludge (50%) and biological sewage sludge (50%) | 5.27^a^ | 0.013 | [2] |
| 3 | 15,000 | 7.37 | 34.7 | Primary sewage sludge (50%) and biological sewage sludge (50%) | 6.35^a^ | 0.016 |  |
| 4 | 4,800 | 7.83 | 37 | Manure | 6.32^a^ | 0.005 | [3] |
| 5 | 8,000 | 8.18 | 40 | Manure | 6.93^a^ | 0.003 |  |
| 6 | 37,500 | 7.75 | 40 | Manure | 5.58^a^ | 0.006 |  |
| 7 | 750 | 7.82 | 52 | Manure | 1.95^a^ | 0.002 |  |
| 8 | 1,400 | 8.18 | 50 | Manure | 21.29^a^ | 0.009 |  |
| 9 | 1,400 | 8.36 | 52 | Manure | 10.74^a^ | 0.003 |  |
| 10 | 2,400 | 8.10 | 52 | Manure | 4.49^a^ | 0.002 |  |
| 11 | 2,400 | 8.10 | 52 | Manure | 1.05^a^ | 0.001 |  |
| 12 | 880 | 8.03 | 53 | Manure | 15.67^a^ | 0.008 |  |
| 13 | 9,000 | 7.01 | 37 | Sewage sludge | 1.19^a^ | 0.007 |  |
| 14 | 5,000 | 7.11 | 37 | Sewage sludge | 0.65^a^ | 0.003 |  |
| 15 | 6,000 | 7.10 | 39 | Sewage sludge | 0.64^a^ | 0.003 |  |
| 16 | 1,400 | 7.33 | 37 | Sewage sludge | 0.60^a^ | 0.002 |  |
| 17 | 670 | 7.53 | 36 | Sewage sludge | 1.70^a^ | 0.003 |  |
| 18 | 9,903 | 7.40 | 35 | Sewage sludge | 2.50^a^ | 0.006 | [4] |
| 19 | 6,568 | 7.20 | 35 | Sewage sludge | 2.83^a^ | 0.010 |  |
| 20 | 1,400 | 6.40 | 35 | Sewage sludge | 2.50^a^ | 0.056 |  |
| 21 | 1,600 | 6.10 | 35 | Sewage sludge | 11.33^a^ | 0.495 |  |
| 22 | Full-scale | 7.20 | 36 | Sewage sludge | 1.00^a^ | 0.004 | [5] |
| 23 | Full-scale | 6.50 | 30 | Brewery industry | 1.00^a^ | 0.018 |  |
| 24 | Full-scale | 7.00 | 44 | Dairy industry | 90.12^a^ | 0.515 |  |
| 25 | Full-scale | 7.50 | 37 | Dairy and fish waste | 1.83^a^ | 0.003 |  |
| 26 | Full-scale | 7.00 | 36 | Sugar industry | 1.00^a^ | 0.006 |  |
| 27 | Full-scale | 7.10 | 35 | Yeast industry | 2.33^a^ | 0.011 |  |
| 28 | 2,700 | 8.18 | 32-39.3 | Sewage sludge | 7.38-27.85^a^ | 0.011 | [6] |
| 29~60 | Full-scale | 7.10-8.50 | 37 and 55 | Primary sludge and surplus activated sludge | 0.5-20 | 0.099 | [7] |
| 61 | 4,000 | 8.30 | 47 | Municipal solid waste | 3.3^a^ | 0.001 | [8] |
| 62 | 4,000 | 8.50 | 47 | Municipal solid waste | 3.92^a^ | 0.001 |  |
| 63 | 1,500 | 8.52 | 38 | Maize, manure | 44.80^a^ | 0.008 |  |
| 64 | 1,500 | 8.24 | 34 | Maize, manure | 0.00^a^ | 0.000 |  |
| 65 | 1,000 | 8.03 | 54 | Slaughterhouse waste | 86.80^a^ | 0.051 |  |
| 66 | 1,000 | 8.03 | 54 | Slaughterhouse waste | 86.80^a^ | 0.051 |  |
| 67 | 2,500 | 8.12 | 34 | Maize, manure | 4.35^a^ | 0.002 |  |
| 68 | 3,600 | 7.52 | 54 | Manure, organic biological waste, energy crops, slaughterhouse waste | 51.64^a^ | 0.098 |  |
| 69 | 3,600 | 7.52 | 54 | Manure, organic biological waste, energy crops, slaughterhouse waste | 59.45^a^ | 0.113 |  |
| 70 | 3,150 | 8.50 | 50 | organic fraction municipal solid waste | 1.02^a^ | 0.000 |  |
| 71 | 3,150 | 8.30 | 52 | organic fraction municipal solid waste | 26.15^a^ | 0.008 |  |
| 72 | 3,450 | 8.20 | 50 | organic fraction municipal solid waste | 0.00^a^ | 0.000 |  |
| 73 | 1,200 | - | 55 | Maize, manure | 18.51^a^ | - |  |
| 74 | 1,200 | - | 55 | Maize, manure | 0.38^a^ | - |  |
| 75 | 1,200 | - | 55 | Maize, manure | - | - |  |
| 76 | 1,200 | - | 42 | Maize, manure | 44.48^a^ | - |  |
| 77 | 1,000 | 8.19 | 34 | Maize, fats, fruit waste | 7.13^a^ | 0.003 |  |
| 78 | 3,255 | 7.35 | 33 | Sludge, manure | 0.00^a^ | 0.000 |  |
| 79 | 4,000 | 7.35 | 34 | Wastewater sludge | 0.00^a^ | 0.000 |  |
| 80 | 4,000 | 7.48 | 34 | Wastewater sludge | 0.00^a^ | 0.000 |  |
| 81 | 4,000 | 7.43 | 34 | Wastewater sludge | 0.00^a^ | 0.000 |  |
| 82 | 1,200 | 8.00 | 34 | Maize, manure | 36.63^a^ | 0.021 |  |
| 83 | 1,200 | 8.06 | 34 | Maize, manure | 34.90^a^ | 0.017 |  |
| 84 | 1,250 | 8.05 | 34 | Manure | 5.57^a^ | 0.003 |  |
| 85 | 2,000 | 7.92 | 54 | Maize, manure | 63.39^a^ | 0.048 |  |
| 86 | 2,000 | 7.86 | 54 | Maize, manure | 61.67^a^ | 0.053 |  |
| 87 | 3,000 | 7.76 | 34 | Maize, manure | 287.19^a^ | 0.287 |  |
| 88 | 3,200 | 8.25 | 34 | Maize, manure | 3.39^a^ | 0.001 |  |
| 89 | 1,500 | 8.02 | 34 | Organic biological waste | 43.70^a^ | 0.024 |  |
| 90 | 1,500 | 8.02 | 34 | Organic biological waste | 176.57^a^ | 0.097 |  |
| 91 | 1,500 | 8.02 | 34 | Organic biological waste | 6.48^a^ | 0.004 |  |
| 92 | Full-scale | 8.30 | 34 | - | 0.00^a^ | 0.000 |  |
| 93 | 1,500 | 8.10 | 34 | Organic biological waste | 1.89^a^ | 0.001 |  |
| 94 | Full-scale | 8.35 | 34 | Manure, organic biological waste | 8.56^a^ | 0.002 |  |
| 95 | Full-scale | 7.12 | 34 | Potato wastewater | 2.54^a^ | 0.011 |  |
| 96 | 274 | 7.14 | 34 | Brewery wastewater | 0.00^a^ | 0.000 |  |
| 97 | 1,210 | 7.19 | 35 | Paper mill wastewater | 1.96^a^ | 0.007 |  |
| 98 | Full-scale | 7.10 | 34 | Potato wastewater | 0.00^a^ | 0.000 |  |
| 99 | Full-scale | 8.02 | 37-40 | Food waste | 15.40 | 0.008 | Data monitored by our group |
| 100 | Full-scale | 7.84 | 37-40 | Food waste | 19.89 | 0.017 |  |
| 101 | Full-scale | 7.89 | 37-40 | Food waste | 19.87 | 0.015 |  |
| 102 | Full-scale | 7.95 | 37-40 | Food waste | 60.63 | 0.039 |  |
| 103 | Full-scale | 7.82 | 37-40 | Food waste | 18.78 | 0.016 |  |
| 104 | Full-scale | 7.98 | 37-40 | Food waste | 5.40 | 0.003 |  |
| 105 | Full-scale | 8.35 | 37-40 | Vegetable waste and expired food | 1.09 | 0.000 |  |
| 106 | Full-scale | 7.40 | 37-40 | Industrial food waste | 44.75 | 0.102 |  |

^a^ VFAs concentration was converted to acetate concentration.

**References**

1. Ike M, Inoue D, Miyano T, Liu TT, Sei K, Soda S, et al. Microbial population dynamics during startup of a full-scale anaerobic digester treating industrial food waste in Kyoto eco-energy project. Bioresour Technol. 2010;101(11):3952-7.

2. Hao L, Bize A, Conteau D, Chapleur O, Courtois S, Kroff P, et al. New insights into the key microbial phylotypes of anaerobic sludge digesters under different operational conditions. Water Res. 2016;102:158-69.

3. Luo G, Fotidis IA, Angelidaki I. Comparative analysis of taxonomic, functional, and metabolic patterns of microbiomes from 14 full-scale biogas reactors by metagenomic sequencing and radioisotopic analysis. Biotechnol Biofuels. 2016;9:51.

4. Shin SG, Koo T, Lee J, Han G, Cho K, Kim W, et al. Correlations between bacterial populations and process parameters in four full-scale anaerobic digesters treating sewage sludge. Bioresour Technol. 2016;214:711-21.

5. Regueiro L, Veiga P, Figueroa M, Alonso-Gutierrez J, Stams AJM, Lema JM, et al. Relationship between microbial activity and microbial community structure in six full-scale anaerobic digesters. Microbiol Res. 2012;167(10):581-9.

6. Shi X, Zhao J, Chen L, Zuo J, Yang Y, Zhang Q, et al. Genomic dynamics of full-scale temperature-phased anaerobic digestion treating waste activated sludge: Focusing on temperature differentiation. Waste Manage. 2019;87:621-8.

7. Kirkegaard RH, McIlroy SJ, Kristensen JM, Nierychlo M, Karst SM, Dueholm MS, et al. The impact of immigration on microbial community composition in full-scale anaerobic digesters. Scientific Reports. 2017;7(1):9343.

8. De Vrieze J, Saunders AM, He Y, Fang J, Nielsen PH, Verstraete W, et al. Ammonia and temperature determine potential clustering in the anaerobic digestion microbiome. Water Res. 2015;75:312-23.
